# *Plasmodium falciparum* SERA5 plays a non-enzymatic role in the malarial asexual blood-stage lifecycle

**DOI:** 10.1111/mmi.12941

**Published:** 2015-02-11

**Authors:** Robert Stallmach, Manoli Kavishwar, Chrislaine Withers-Martinez, Fiona Hackett, Christine R Collins, Steven A Howell, Sharon Yeoh, Ellen Knuepfer, Avshalom J Atid, Anthony A Holder, Michael J Blackman

**Affiliations:** 1 Division of Parasitology, MRC National Institute for Medical ResearchLondon, NW7 1AA, UK; 2 Division of Molecular Structure, MRC National Institute for Medical ResearchLondon, NW7 1AA, UK

## Abstract

The malaria parasite *Plasmodium falciparum* replicates in an intraerythrocytic parasitophorous vacuole (PV). The most abundant *P. falciparum* PV protein, called SERA5, is essential in blood stages and possesses a papain-like domain, prompting speculation that it functions as a proteolytic enzyme. Unusually however, SERA5 possesses a Ser residue (Ser596) at the position of the canonical catalytic Cys of papain-like proteases, and the function of SERA5 or whether it performs an enzymatic role is unknown. In this study, we failed to detect proteolytic activity associated with the Ser596-containing parasite-derived or recombinant protein. However, substitution of Ser596 with a Cys residue produced an active recombinant enzyme with characteristics of a cysteine protease, demonstrating that SERA5 can bind peptides. Using targeted homologous recombination in *P. falciparum*, we substituted Ser596 with Ala with no phenotypic consequences, proving that SERA5 does not perform an essential enzymatic role in the parasite. We could also replace an internal segment of SERA5 with an affinity-purification tag. In contrast, using almost identical targeting constructs, we could not truncate or C-terminally tag the *SERA5* gene, or replace Ser596 with a bulky Arg residue. Our findings show that SERA5 plays an indispensable but non-enzymatic role in the *P. falciparum* blood-stage life cycle.

## Introduction

Malaria is the biggest killer of all the parasitic diseases. Of the five malaria species that infect humans, the deadliest is *Plasmodium falciparum*. A number of effective antimalarial drugs are currently in clinical use, but the continued appearance and spread of drug-resistant *P. falciparum* strains is an acute threat. As a result, antimalarial drugs with new modes of action are urgently needed for use in new prophylactic or therapeutic combinations (Burrows *et al*., 2013; Ashley *et al*., 2014; White *et al*., 2014). All the clinical manifestations of malaria arise from the asexual blood stages of the parasite life cycle, where at each round of intraerythrocytic growth, the parasite divides inside a membrane-bound parasitophorous vacuole (PV) to form a multinucleated schizont. Upon rupture of the infected cell, 16 or more daughter merozoites are released to invade fresh erythrocytes and repeat the cycle. It has long been recognised that merozoite egress and subsequent erythrocyte invasion require the activity of parasite proteases, and several protease inhibitors prevent these crucial steps in the parasite life cycle (for a review, see Blackman, 2008). Compounds that directly target proteolytic enzymes form the basis of highly effective drugs against important pathogens including HIV and hepatitis C virus, so inhibitors of essential malarial proteases may similarly provide routes to the development of novel antimalarials.

SERA5 (PlasmoDB ID: PF3D7_0207600) is the prototypic member of a family of nine proteins in *P. falciparum* that share a similar overall structure which includes a central module with homology to papain-like (clan CA, family C1) cysteine peptidases. Whilst SERA6, 7 and 8 possess a canonical catalytic Cys residue in their putative active site, this residue is replaced by Ser in SERA5 and all other *P. falciparum* SERA family members (Arisue *et al*., 2011). Despite this unusual feature, the common possession of a papain-like domain has engendered much speculation that all the SERA proteins are peptidases. Gene disruption studies have suggested that whereas most *SERA* genes are dispensable for asexual blood stage development, those that encode SERA5 and SERA6 – which are both relatively abundant, soluble proteins that localise to the PV – appear essential in blood stages (Miller *et al*., 2002; McCoubrie *et al*., 2007; Ruecker *et al*., 2012). SERA5 undergoes extensive proteolytic processing at around the time of parasite egress, and a number of studies have linked this processing and SERA function to egress (Delplace *et al*., 1988; Pang *et al*., 1999; Li *et al*., 2002). Initial processing of SERA5 is mediated by a parasite subtilisin-like serine protease called SUB1 (Yeoh *et al*., 2007; Withers-Martinez *et al*., 2014), which is discharged into the PV just minutes before egress (Collins *et al*., 2013a). Cleavage by SUB1 at two (or three) positions converts full-length SERA5 to a 47 kDa N-terminal fragment (P47) and a C-terminal 18 kDa fragment (P18), releasing a central 56 kDa species (P56) that contains the papain-like module. P56 is further trimmed at an unknown site near its C-terminal end by an unidentified additional proteolytic activity (referred to here as protease X) resulting in a 50 kDa fragment called P50 (Debrabant *et al*., 1992). P50 is released upon schizont rupture as a fully soluble protein, whereas P47 and P18 remain in a disulphide-bonded heterodimeric complex that selectively binds to the released merozoite surface (Li *et al*., 2002). Consistent with this, antibodies against P47 – but not against the central domain of SERA5 – inhibit merozoite invasion of erythrocytes (Barr *et al*., 1991; Sugiyama *et al*., 1996; Fox *et al*., 1997; Pang *et al*., 1999), and several reports have indicated that an immune response to SERA5 can protect against parasite replication *in vivo* (e.g. Inselburg *et al*., 1991; Enders *et al*., 1992; Horii *et al*., 2010). Furthermore, epidemiological and vaccine-based studies have shown that antibodies to SERA5 are readily induced by natural infection or experimental vaccination (Aoki *et al*., 2002; Okech *et al*., 2006; Horii *et al*., 2010). Proteolytic processing of SERA5 at merozoite egress has been proposed to represent a zymogen activation event, perhaps converting SERA5 to a functional papain-like enzyme bearing the catalytic centre residue of a serine peptidase (Blackman, 2008). However, there is only limited evidence for this, and the question of whether SERA5 functions as a protease or peptidase in the parasite has been a matter of considerable debate. A recombinant form of the papain-like domain of SERA5 was reported to display low-level chymotrypsin-like activity *in vitro* (Hodder *et al*., 2003), but that study did not control for the possible presence of low-level contaminating proteases. A more recent study appeared to confirm the peptidase activity of the same recombinant protein (Kanodia *et al*., 2014). Protein- or peptide-based reagents designed to target the papain-like module of SERA5 were shown to have growth inhibitory activity against *P. falciparum in vitro* (Fairlie *et al*., 2008; Alam and Chauhan, 2012; Kanodia *et al*., 2014), consistent with the notion that the papain-like domain of SERA5 has an important physiological function. In apparent conflict with these data, X-ray crystallographic analysis of the SERA5 papain-like domain revealed features that cast doubt on its capacity to interact with peptide substrates in a canonical manner (Hodder *et al*., 2009). It remains to be established whether SERA5 has an essential enzymatic role in the parasite.

This study directly addresses the question of whether SERA5 possesses enzymatic activity pivotal for *P. falciparum* viability in asexual blood stages. We show that while SERA5 does not have detectable protease activity *in vitro* in our hands, replacement of its putative catalytic Ser (Ser596) with a Cys residue creates an active peptidase, proving that SERA5 is capable of binding peptides in a substrate-like manner. Using genetic modification of *P. falciparum*, we go on to prove that SERA5 does not perform an important enzymatic role in the parasite, but confirm that the protein is nonetheless indispensable for blood-stage viability. We speculate that SERA5 plays an essential but non-catalytic regulatory role in the protease-mediated pathway that leads to parasite egress from host red blood cells.

## Results

### Mapping the protease X cleavage site shows that the terminal SERA5 P50 processing product includes the entire papain-like domain of SERA5

**Figure 1:**
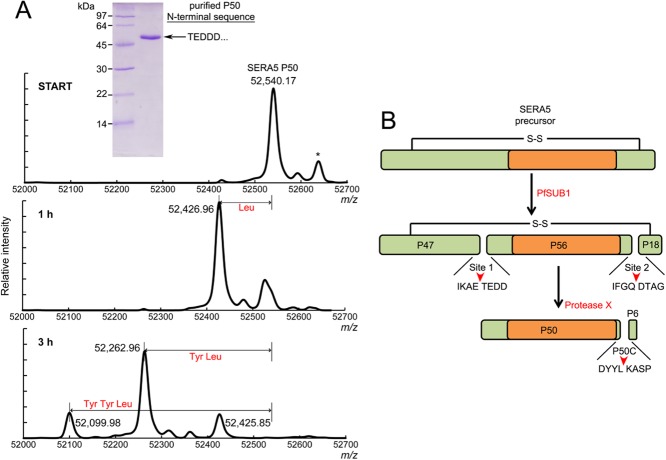
N- and C-terminal mapping of purified P50 shows that it contains the entire papain-like domain of SERA5.A. N-terminal sequencing and electrospray mass spectrometry of intact purified P50 prior to (START panel) or after limited digestion with carboxypeptidase Y (1 h and 3 h panels). Shifts in the *m*/*z* values are evident corresponding to the loss of a Leu residue and two Tyr residues from the C-terminus. The inset shows purified, SDS–PAGE fractionated P50 stained with Coomassie Blue. Only the first five N-terminal residues were established by Edman degradation.B. Schematic depicting the two-step processing of SERA5. Cleavage of full-length SERA5 by PfSUB1 at sites 1 and 2 flanking the papain-like domain (orange) releases P56. This is subsequently trimmed near its C-terminus by protease X, which cleaves at the P50C site identified in this study. This is predicted to release a 44-residue peptide (which has not been isolated) here termed P6, leaving the entire papain-like domain encompassed within the final P50 processing product. Note that, in some allelic forms of SERA5, PfSUB1 also cleaves at an allele-specific third position called site 3, within the N-terminal (P47) segment of the SERA5 precursor (Li *et al*., 2002; Yeoh *et al*., 2007). For clarity, this is not shown here.

Proteolytic processing of SERA5 during egress results in release of the P50 processing product in fully soluble form. Since SERA5 is a highly abundant *P. falciparum* protein, we decided that for initial investigations of its potential protease activity, we would examine native, parasite-derived P50 purified from *in vitro* cultures. Supernatants collected from synchronised, high parasitaemia schizont cultures that had been allowed to undergo egress overnight were fractionated by Fast Protein Liquid Chromatography (FPLC) ion exchange and gel filtration chromatography. The resulting preparations, which contained microgram amounts of P50 protein, were chromatographically homogeneous with no sign of co-purifying proteins (Fig. 1A inset). Importantly, in the final gel filtration step of the purification regimen (not shown), all the detectable P50 eluted at a position in accord with its estimated monomeric mass (see discussion later), suggesting that it does not multimerise or form a stable stoichiometric complex with a partner protein. Edman degradation confirmed the N-terminus of the purified P50 as Thr391, as previously shown (Debrabant *et al*., 1992), consistent with cleavage by PfSUB1 at the known site 1 position 387IKAE↓TEDD394 (Yeoh *et al*., 2007). It was reasoned that the potential for the purified protein to display protease activity is likely to depend on it containing the entire papain-like domain, so to address whether this was the case, the purified P50 was analysed in detail to determine the extent of its primary structure. Electrospray mass spectrometry provided a mass for the non-reduced intact protein of 52 540.17 Da (Fig. 1A, START panel). The central region of SERA5 contains a total of 12 Cys residues, all of which are known (Hodder *et al*., 2009) or expected to be engaged in intramolecular disulfide bonds, so this experimentally determined mass closely corresponds to the SERA5 primary sequence extending from Thr391 to Leu842 (predicted mass in non-reduced form 52 540.60). To confirm this, we sought to identify the C-terminal residue of P50. Purified protein was subjected to controlled digestion with carboxypeptidase Y, an exopeptidase which cleaves single residues specifically from the C-terminus of polypeptides. Mass spectrometric analysis of samples of the digestion time course revealed time-dependent shifts in the mass of the treated P50 consistent with the sequential loss of a Leu (or Ile) and two Tyr residues (Fig. 1A, 1h and 3h). A Tyr-Tyr-Leu motif exists at only one position in the SERA5 primary sequence (Supplementary Fig. S1), and this is preceded by an Asp residue, known to be removed relatively inefficiently by carboxypeptidase Y (Hayashi *et al*., 1973). Collectively, these results are fully consistent with the C-terminal residue of P50 being Leu842, mapping the protease X cleavage site (hereafter referred to as the P50C site) to the motif 839DYYL↓KASP846 which lies 44 residues upstream of the PfSUB1 cleavage site 2 (Fig. 1B and Supplementary Fig. S1). The predicted papain-like domain of SERA5 extends approximately from Ser558-Lys832 (Hodder *et al*., 2009), so these data conclusively show that the P50 processing product includes the entire papain-like module of SERA5 (Fig. 1B).

### Purified parasite-derived SERA5 P50 displays no detectable protease activity

Having shown that P50 encompasses sufficient primary sequence to contain all the structural elements of the SERA5 papain-like fold, the purified parasite protein was subjected to a range of assays designed to detect peptidase activity. Incubation in solution with a number of unlabelled or fluorogenic peptidyl substrates (listed in Supplementary Table S1) or with a range of heat-denatured proteins (not shown), under a range of buffer conditions, failed to detect any peptidase activity *in trans* or any tendency of the P50 to degrade upon storage (which might indicate autolytic protease activity) (data not shown). Xymogram analysis using denatured gelatin or bovine serum albumin (BSA) as in-gel substrates similarly detected no protease activity associated with the purified P50, and attempts to label the purified protein with the activity-based serine hydrolase-reactive probes FP biotin and JCP104 also failed (not shown). These findings suggested that either native P50 lacks peptidase activity or that its requirements for activity are highly stringent.

### Recombinant SERA5 has detectable peptidase activity only when Ser596 is replaced by Cys

**Figure 2:**
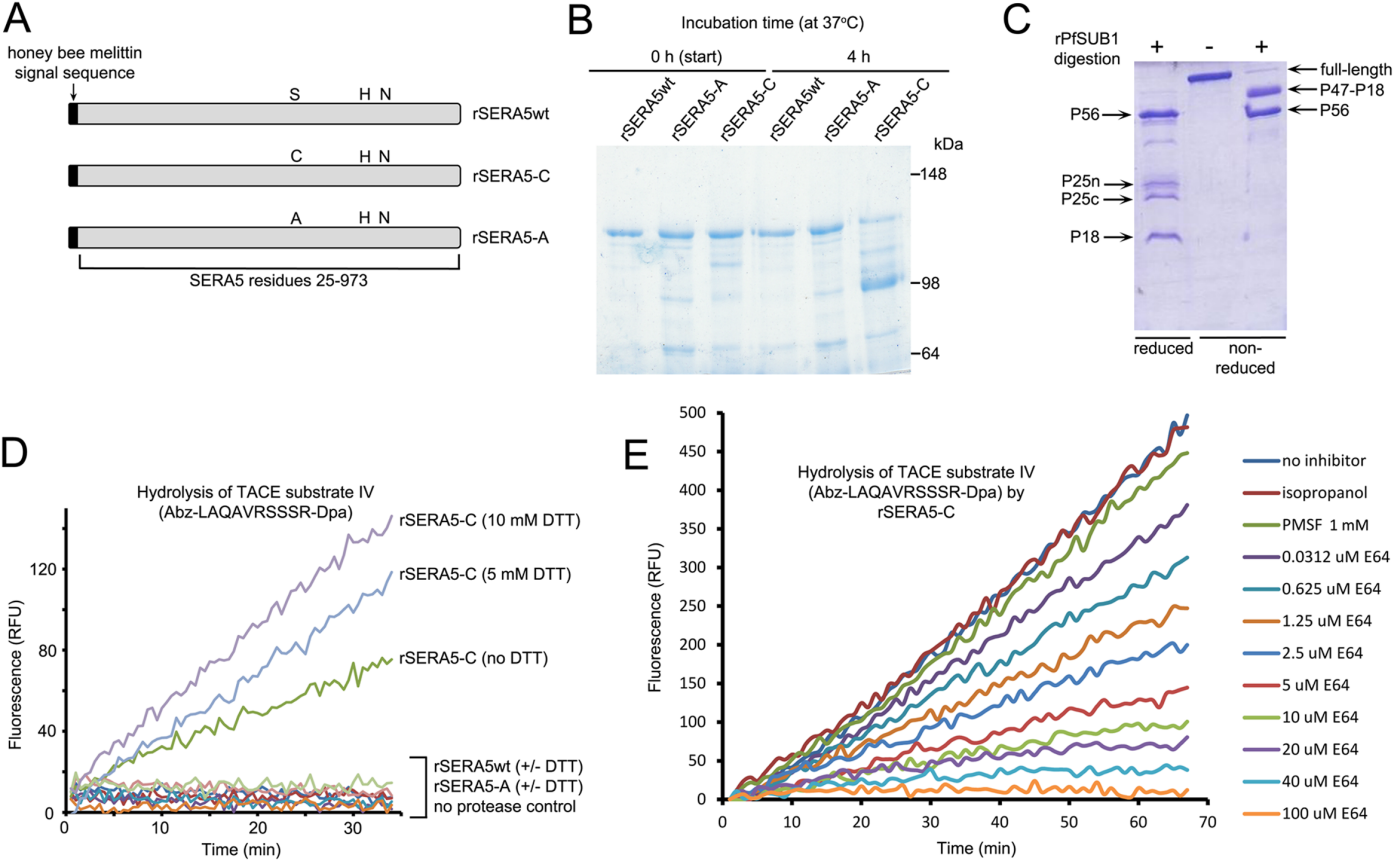
Recombinant SERA5 containing a Ser596Cys mutation exhibits proteolytic activity.A. Schematic depicting the three recombinant forms of full-length SERA5 produced in this study. The endogenous SERA5 secretory signal sequence (Met1-Gly24) was replaced by that of honey bee melittin in order to optimise secretion from baculovirus-infected Tn5 cells. The relative positions of the putative catalytic triad residues Ser596, His762 and Asn787 are indicated, as are the Ala and Cys residues that replace Ser596 in rSERA5-A and rSERA5-C respectively.B. Purified recombinant SERA5-C is associated with an autolytic protease activity. Preparations of insect cell-derived rSERA5wt, rSERA5-A and rSERA5-C, expressed and purified under identical conditions, were analysed by SDS–PAGE and Coomassie staining either immediately following purification (0 h) or following further incubation at 37°C for 4 h. All three protein samples were purified in a partially degraded form, but whereas rSERA5wt and rSERA5-A were completely stable to further incubation, purified rSERA5-C displayed substantial degradation to a ∼ 100 kDa form. Each track contains approximately 2 μg of protein.C. Correct processing of rSERA5wt by rPfSUB1. Purified full-length rSERA5wt (central lane; ∼ 3 μg loaded) was exhaustively digested with rPfSUB1 and subjected to SDS–PAGE under reducing (left-hand lane) or non-reducing (right-hand lane) conditions. Indicated are the expected products of digestion at sites 1 and 2 (Yeoh *et al*., 2007), as well as of cleavage within the allele-specific site 3 within P47 to produce the P25n and P25c fragments (Li *et al*., 2002). Cleavage at the predicted sites was confirmed by N-terminal sequence analysis of the reduced digestion products (not shown).D. Typical progress curves showing cleavage of fluorogenic substrate Abz-LAQAVRSSSR-Dpa (10 μM) by rSERA5-C (see *Experimental procedures* section for additional details of assay conditions). Cleavage was enhanced in the presence of DTT. No hydrolysis of this or any of the other peptide substrates tested (see Supplementary Table S1) was observed in the presence of equivalent concentrations of rSERA5wt or rSERA5-A.E. Typical progress curves showing that cleavage of Abz-LAQAVRSSSR-Dpa (10 μM) by rSERA5-C is highly sensitive to the cysteine protease inhibitor E64 (used at the range of concentrations indicated), but not the serine protease inhibitor PMSF (1 mM). Isopropanol (1% v/v) was used as a control for the solvent in which the PMSF was dissolved.

The catalytic activity of papain-like peptidases is critically dependent upon a triad of residues which possesses at its core a nucleophilic Cys residue. Experimental substitution of this canonical Cys in archetypal cysteine proteases invariably results in substantial reduction or ablation of enzyme activity (e.g. Clark and Lowe, 1978; Coulombe *et al*., 1996). In the light of our inability to demonstrate peptidase activity associated with the native P50 protein, we speculated that replacement of its predicted catalytic Ser596 with a Cys residue might confer hydrolytic activity on the protein or enhance any existing activity not detected by our experimental protocols. To test this idea, we generated constructs designed for the recombinant expression of full-length SERA5 in wild-type form (called rSERA5wt), or with the Ser596 residue replaced with a Cys (called rSERA5-C). As a further control for these experiments, we also generated a third construct (called rSERA5-A) designed to replace the Ser596 side chain with an Ala, the methyl side chain of which lacks the nucleophilic Ser Oγ atom or Cys thiol required to attack the carbonyl carbon at the scissile bond of the substrate. The rSERA5-A recombinant product was therefore predicted to entirely lack catalytic activity (Fig. 2A). To ensure correct folding of the recombinant proteins, expression was performed in recombinant baculovirus-infected insect cells, and the recombinant proteins were directed for optimal secretion by replacing the endogenous N-terminal secretory signal sequence with that of honey bee melittin. All three recombinant SERA5 proteins were expressed and purified in similarly high yield (> 20 mg purified protein/litre of insect cell growth medium). Intriguingly, whilst rSERA5wt and rSERA5-A were consistently purified as predominantly full-length proteins, the rSERA5-C variant expressed and purified under identical conditions was reproducibly obtained in a partially degraded form (Fig. 2B). This distinction became more evident upon further incubation of the concentrated purified proteins at 37°C, when extensive conversion of rSERA5-C to a dominant ∼ 100 kDa species took place (Fig. 2B). In contrast, both the rSERA5wt and rSERA5-A proteins were virtually completely stable under the same conditions. Since the proteins had been expressed and purified under identical conditions, it was considered highly unlikely that the observed degradation of the rSERA5-C was due to contaminating proteases (e.g. derived from insect cells). This interpretation was supported by co-incubation of a mixture of the rSERA5-A and rSERA5-C proteins, when again degradation only of the rSERA5-C form was apparent (Supplementary Fig. S2). These results were highly suggestive of an autolytic (*in cis*) protease activity specifically associated with the rSERA5-C mutant.

The recombinant SERA5 proteins were fully soluble and monodisperse as assessed by dynamic light scattering (not shown). To assess their structural integrity, the purified rSERA5wt and rSERA5-A proteins were treated *in vitro* with purified recombinant PfSUB1 (rPfSUB1). This resulted as expected in limited cleavage of the recombinant proteins, producing a digestion pattern (Fig. 2C) consistent with cleavage at the three known PfSUB1 recognition sites in the 3D7 SERA5 sequence (Li *et al*., 2002; Yeoh *et al*., 2007); correct cleavage was confirmed by N-terminal amino acid sequencing of the rSERA5wt digestion fragments (not shown). Peptide cleavage and zymogram assays were then used to examine all three recombinant SERA5 protens for peptidase activity *in trans*, before or following treatment with rPfSUB1 to mimic the first stages of *in vivo* processing of SERA5. No activity was observed in zymogram assays, and no hydrolysis of most of the peptide substrates listed in Supplementary Table S1 was detected under a range of buffer conditions (data not shown). Importantly, the substrates tested included the chymotrypsin substrate succinyl-LLVY-AMC, previously reported to be hydrolysed by an *Escherichia coli*-derived recombinant form of the SERA5 papain-like domain (Fairlie *et al*., 2008; Hodder *et al*., 2009). However, we found one fluorogenic substrate, Abz-LAQAVRSSSR-Dpa (TNFα-converting enzyme substrate IV), to be specifically hydrolysed upon incubation with rSERA5-C (Fig. 2D). No cleavage of this substrate was observed in the presence of intact or rPfSUB1-digested rSERA5-A or rSERA5wt. Importantly, hydrolysis of Abz-LAQAVRSSSR-Dpa in the presence of rSERA5-C was enhanced by low concentrations of the reducing agent dithiothreitol (DTT) (Fig. 2D), and was highly sensitive to the cysteine protease inhibitor *trans*-epoxysuccinyl-L-leucylamido(4-guanidino)butane (E64) with an apparent IC50 of 2.9 ± 0.3 μM, but was insensitive to the chelating agent ethylenediaminetetraacetate (EDTA) and the serine protease inhibitor phenylmethylsulfonyl fluoride (PMSF, 1 mM) (Fig. 2E). Notably, at the highest concentrations of E64 used in these experiments (20, 40 and 100 μM), the progress curves showed a clear biphasic profile, reaching a plateau after 10–40 min, consistent with an irreversible mode of inhibition, as expected (Fig. 2E). The hydrolytic activity associated with rSERA5-C therefore displayed characteristics typical of papain-like cysteine peptidases. Pre-treatment of rSERA5-C with rPfSUB1 had no inhibitory or stimulatory effect on its activity against Abz-LAQAVRSSSR-Dpa (not shown). Mass spectrometric analysis of the dominant digestion product obtained following extended incubation of rSERA5-C with Abz-LAQAVRSSSR-Dpa showed that cleavage took place predominantly at the Arg-Ser bond (Supplementary Fig. S3). Kinetic analyses of initial hydrolysis rates indicated Michaelis–Menten kinetics and allowed determination of an apparent *K*_m_ of ∼ 11.3 μM (Supplementary Fig. S4). No attempts were made to determine *k*_cat_ values since the instability of the rSERA5-C protein described earlier made it difficult to accurately determine concentrations of protein in the preparations used, and active-site titration with E64 was not attempted. Collectively, our observation that a single Ser-to-Cys amino acid substitution in the putative catalytic site can convert wild-type SERA5 into an active enzyme with the characteristics of a cysteine peptidase indicates that the papain-like domain of SERA5 has the capacity to bind peptides in a substrate-like manner. However, our inability under identical conditions to detect peptidase or protease activity in rSERA5wt, and the fact that it displayed no detectable enhanced hydrolytic activity compared with the rSERA5-A mutant, strongly suggests that wild-type SERA5 is not a catalytically active peptidase.

### Targeted mutagenesis demonstrates that SERA5 is not an essential enzyme in *P. falciparum* asexual blood stages

**Figure 3:**
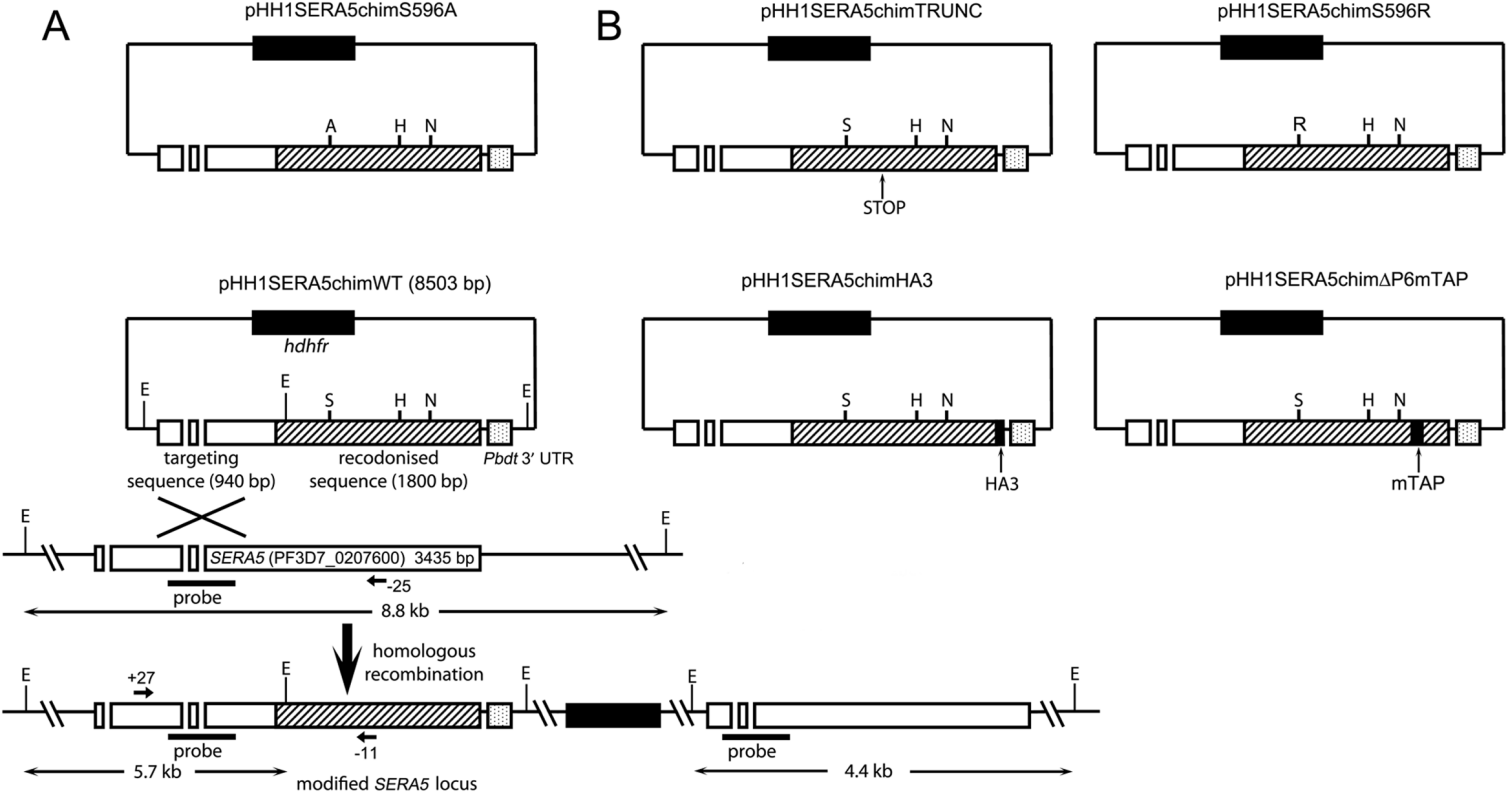
Strategy for modification of the *P. falciparum* *SERA5* locus by single crossover homologous recombination.A. Schematic overview of the pHH1SERA5chimWT and pHH1SERA5chimS596A transfection constructs mentioned in the text, and the expected homologous recombination event at the *SERA5* locus. Each targeting construct contains 940 bp of authentic *SERA5* targeting sequence (open boxes and gaps indicating exon and intron sequences respectively), fused in-frame to 1800 bp of the recodonised *SERA5_synth_* sequence (hatched) in order to select for recombination upstream of the recodonised sequence. The *P. berghei dhfr* 3′ UTR (*Pbdt* 3′ UTR, dotted box) lies just downstream of the *SERA5_synth_* sequence in the plasmids, to regulate termination and polyadenylation of the chimeric gene transcript. The *hdhfr* gene cassette within each targeting construct confers resistance to the antifolate drug WR99210, used for selection of integrants. The relative position of putative catalytic Ser, His and Asn codons are indicated (S, H, N), as well as the mutant Ser596Ala codon (A) in pHH1SERA5chimS596A. Positions of EcoRV restriction enzyme sites (E) used for Southern blot analysis are also indicated, with expected fragment sizes. Positions of hybridisation of primers +27 −11, and −25, used for diagnostic PCR analysis of the unmodified and modified loci, are indicated by small horizontal arrows. Predicted sizes of genomic amplicons obtained with +27 plus −11, and +27 plus −25, are 1911 bp and 1737 bp respectively. The position of the probe used for Southern analysis is indicated.B. Schematic diagrams of the four other transfection constructs used in this study, all derived from pHH1SERA5chimWT by incorporation of the indicated modifications in the *SERA5_synth_* segment. In every other regard, these constructs were identical to pHH1SERA5chimWT.

In view of the results discussed earlier, we reasoned that a direct way to address the question of whether SERA5 performs an essential enzymatic role in the malaria parasite would be to examine the phenotypic consequences of genetically modifying the endogenous single-copy *SERA5* gene so as to ablate any possible catalytic activity. To do this, we used derivatives of a previously described construct (Collins *et al*., 2013b) designed to modify the *SERA5* locus by single-crossover targeted homologous recombination. Briefly, the parental construct contained a targeting element comprising 940 bp of authentic *SERA5* sequence fused in frame to 1800 bp of a recodonised synthetic *SERA5* gene (called *SERA5_synth_*) encoding the remaining 3′ region of the *SERA5* open reading frame (ORF). Integration of this control construct (called pHH1SERA5chimWT) was designed to simply replace the *SERA5* ORF with a chimeric gene encoding the wild-type amino acid sequence, effectively reconstituting the gene (Fig. 3A). In contrast, mutant construct pHH1SERA5chimS596A was designed to substitute the *SERA5* Ser596 codon with an Ala codon upon integration. Otherwise, this construct was identical to pHH1SERA5chimWT. As previously described (Collins *et al*., 2013b), parasites independently transfected by electroporation with both constructs were subjected to repeated cycles of treatment with the antifolate drug WR99210 to select for integrant parasites (a process referred to as drug cycling).

**Figure 4:**
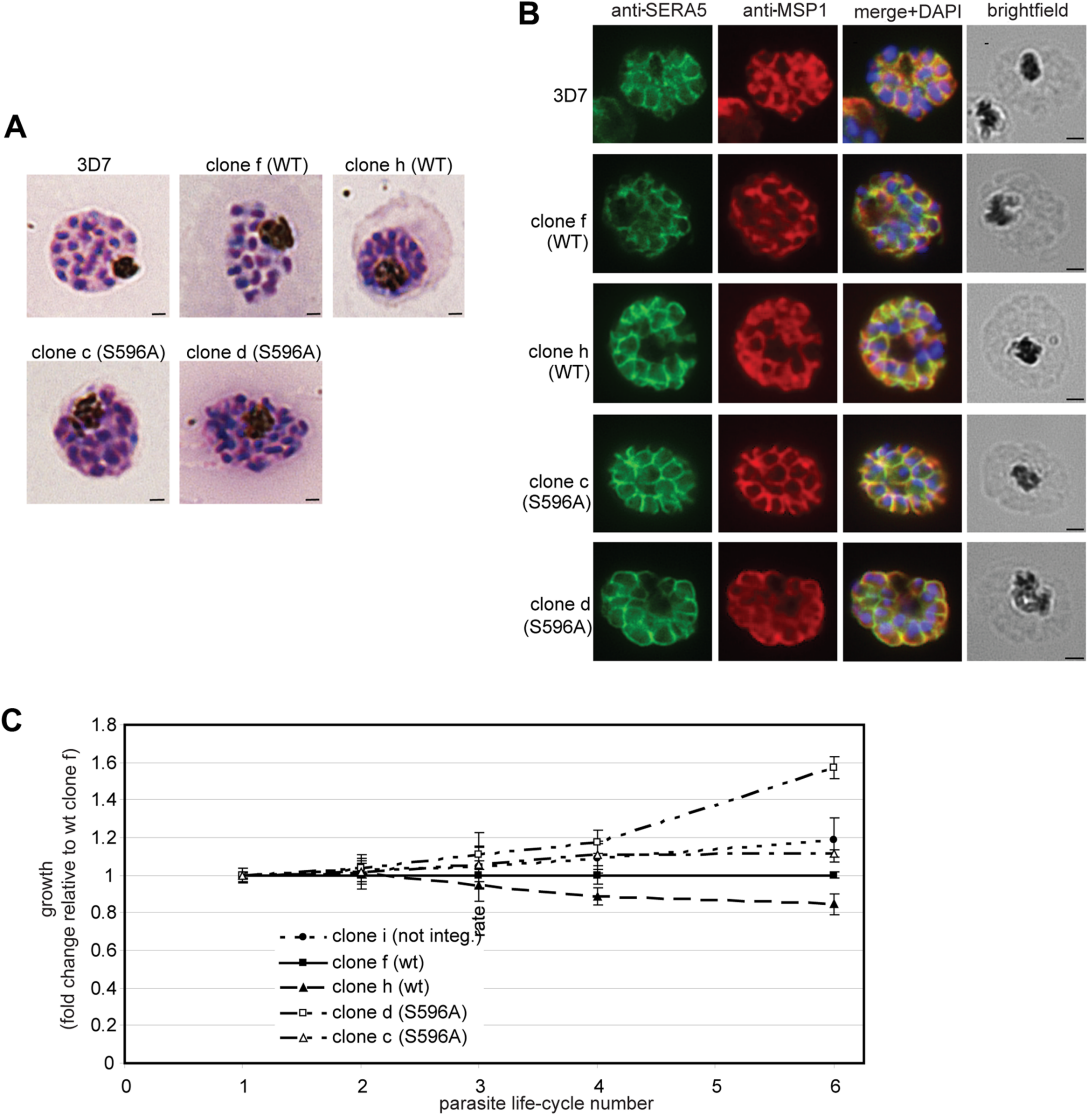
Ala substitution of the ‘catalytic’ Ser596 of SERA5 has no effect on growth or morphology of *P. falciparum* asexual blood stages.A. Images of Giemsa-stained schizonts of the parental 3D7 clone and the indicated transgenic chimWT and chimS596A clones. No discernible morphological differences were evident between the parental and transgenic parasites. Scale bar, 1 μm.B. IFA images of schizonts of the indicated transgenic and parental 3D7 clones, probed with a polyclonal rabbit antiserum raised against full-length rSERA5wt (green) or the anti-MSP1 monoclonal antibody (mAb) 89.1 (red). The merged images include the fluorescence signal of the nuclear stain DAPI. No morphological differences were evident between the parental and transgenic parasites. Scale bar, 1 μm.C. Growth assay. The indicated transgenic clones were synchronised, adjusted to equal parasitaemia levels (0.5%) in triplicate wells and cultured in medium containing WR99210 for a total of six erythrocytic growth cycles (12 days). To determine relative growth rates, parasitaemia was determined at cycles 2, 3, 4 and 6 by FACS analysis as described in *Experimental procedures* section, and is expressed in the plot as fold change in parasitaemia at the indicated time points relative to the chimWT clone f. Error bars indicate SD values of triplicate measurements. The chimS596A clones showed no discernible growth defect compared with the chimWT clones.

Following just a single drug cycle, polymerase chain reaction (PCR) analysis (not shown) showed that, in three out of three independent transfection experiments, both pHH1SERA5chimWT and mutant construct pHH1SERA5chimS596A had efficiently integrated into the *SERA5* locus in the expected manner. By drug cycle 4, Southern blot analysis of the six uncloned parasite lines, called chimWT 1–3 and chimS596A 1–3 respectively, indicated virtually quantitative integration of both constructs in five out of six cases (Supplementary Fig. S5A). To study the integrant parasites in detail, parasite clones were derived by limiting dilution from two each of the chimWT and chimS596A lines. No differences were observed in the rate of outgrowth of these clones, providing an initial indication that the Ser596Ala mutation did not affect parasite viability (not shown, but see results later). Southern blot analysis of 10 clones (Supplementary Fig. S5B) confirmed that integration of the transfection constructs into the *SERA5* locus had occurred in the expected manner in all but one pHH1SERA5chimWT-transfected clone (called clone i, which appeared to stably harbour the non-integrated input plasmid, and was subsequently used as a control in PCR experiments). Microscopic examination of fixed, Giemsa-stained parasites from the clones detected no discernible morphological differences between them (Fig. 4A), whilst immunofluorescence analysis (IFA) showed normal expression and distribution of SERA5 in all clones (Fig. 4B). Detailed examination of the growth rates of the individual clones over six cycles of erythrocytic growth confirmed that there was no growth defect associated with the Ser596Ala substitution (Fig. 4C).

The *P. falciparum SERA5* gene comprises four exons, two of which are completely encompassed by the sequence contained within the targeting region of the pHH1SERA5chimWT and pHH1SERA5chimS596A integration constructs (Fig. 3A). In the light of the unexpected results discussed earlier, we considered the possibility that following integration of the transfection plasmid, and in spite of the presence of the *Pbdt* 3′ UTR sequence located immediately downstream of the chimeric *SERA5* ORF, run-through transcription could potentially occur across the entire modified locus. Were this to occur, a splice event between one of the duplicated introns (arising from partial duplication of the native *SERA5* sequence in the modified locus; see Fig. 3A) could allow excision of an extended cryptic intron (including the integrated plasmid backbone) from this large RNA transcript, resulting in SERA5 expression from a reconstituted native mature mRNA rather than from the chimeric ORF as intended. To investigate this unlikely possibility, reverse transcription-PCR analysis was performed on mRNA isolated from selected transgenic clones, using the same primer pairs used to assess integration at the genomic level. The results (Supplementary Fig. S6) showed that only chimeric cDNA could be amplified from the transgenic parasites, convincingly ruling out this hypothetical possibility. In the case of the chimS596A clone examined, the presence of the Ala596 codon in the amplified cDNAs was confirmed by restriction enzyme digest using a BstU1 site diagnostic of the modified codon (Supplementary Fig. S6B). Collectively, the normal morphology and growth of the transgenic chimS596A parasite clones strongly suggested that SERA5 does not perform an essential enzymatic activity in *P. falciparum* asexual blood stages.

### The Ser596Ala substitution in SERA5 does not result in up-regulation of expression of SERA5 or other SERA family members

**Figure 5:**
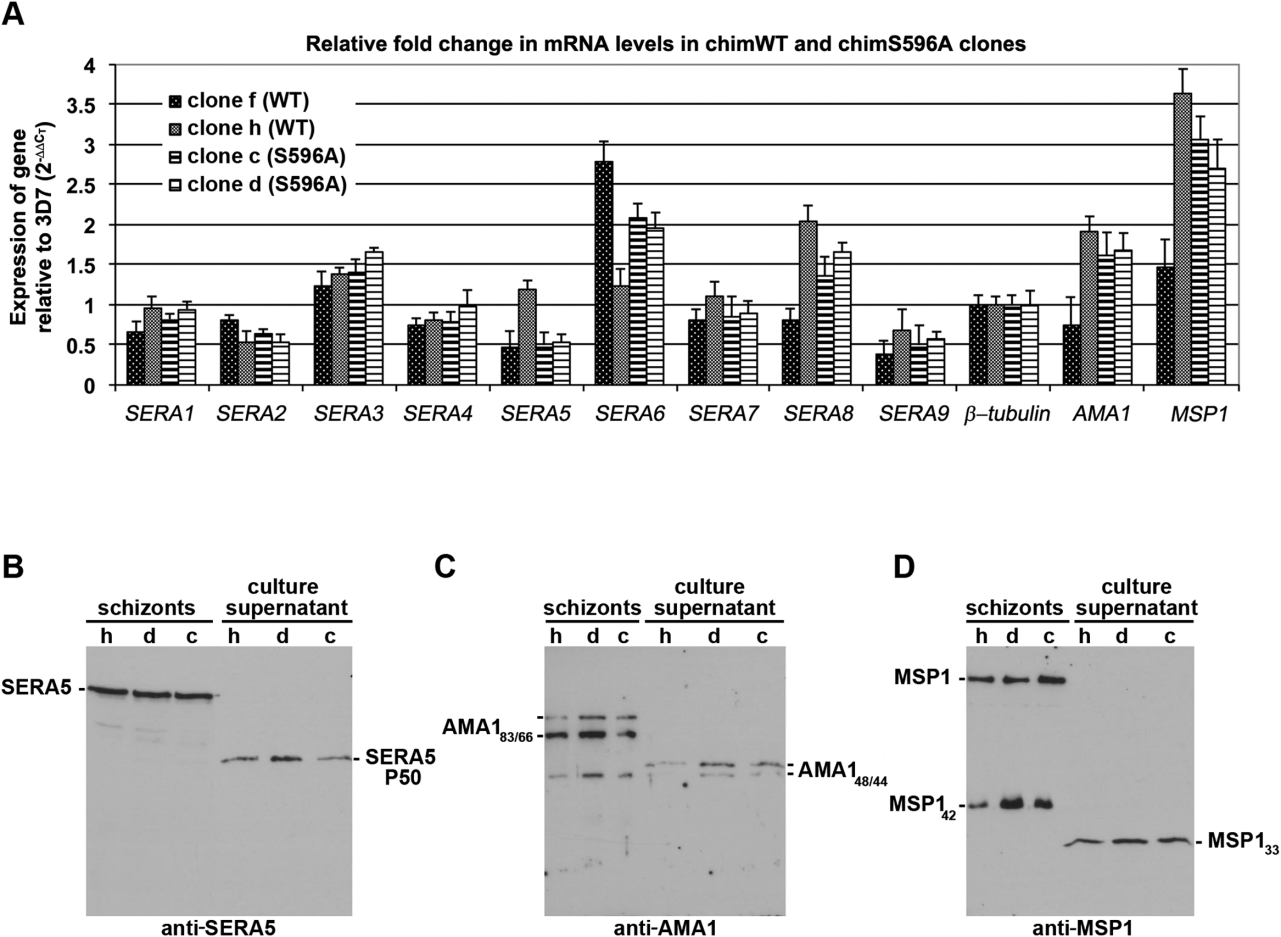
No detectable change in expression of *SERA5* or other *SERA* family members in response to the Ser596Ala substitution.A. SYBR Green-based real-time two-step RT-qPCR analysis of RNA isolated from the indicated transgenic parasite clones was performed in triplicate using primers specific for all nine *SERA* family members, as well as the single-copy *AMA1*, *MSP1* and *β-tubulin* genes, as described in *Experimental procedures* section. Gene transcript levels were normalised to the *β-tubulin* signal in each clone and compared with the respective levels in the parental 3D7 clone. Data are expressed as relative fold difference in mRNA levels compared with the parental 3D7 clone, with error bars indicating range values. No substantive alteration in expression levels of any of the *SERA* genes were observed in the chimS596A parasites compared with their chimWT counterparts.B. Comparison by Western blot of SERA5 protein levels in schizont extracts and culture supernatants of clone h (chimWT) and the two chimS596A clones d and c. Blots were probed with the anti-SERA5 mAb 24C6.1F1. Note that the schizont extracts contain predominantly full-length SERA5, whilst the culture supernatants contain predominantly processed P50, as expected.C and D. Loading controls for panel B. Blots produced using exactly the same loading volumes as in B were probed with a polyclonal rabbit antiserum anti-AMA1 serum (C) or the anti-MSP1 human mAb X509 (D). The various proteolytically processed forms of schizont-derived and supernatant-derived AMA1 and MSP1 are indicated.

As mentioned in the Introduction, *SERA5* is a member of a family of nine homologous genes in *P. falciparum*, six of which (*SERA1–5* and *SERA9*) possess a Ser codon in the position of the putative catalytic residue. Previous work from Crabb and colleagues (Miller *et al*., 2002; McCoubrie *et al*., 2007) consistently failed to isolate parasites harbouring a disrupted *SERA5* gene, suggesting that the function of SERA5 cannot be assumed by any other member(s) of the family in asexual blood stages. Despite this, we considered it important to address the possibility that, were SERA5 function to have been compromised in the transgenic chimS596A parasites described earlier, a compensatory up-regulation of either the modified *SERA5* gene or of other members of the gene family might have occurred. If so, this could potentially explain the absence of an obvious phenotype. To address this possibility, we compared expression of all members of the *SERA* family in selected chimWT and chimS596A clones at the RNA level. To do this, we performed RT-qPCR to monitor the expression levels of all nine *SERA* genes, normalising against the *β-tubulin* gene and using the *AMA1* and *MSP1* genes as stage-specific expression marker genes. No significant up-regulation of transcription of *SERA5* or any other *SERA* gene in the chimS596A clones relative to their chimWT counterparts was detected by this analysis (Fig. 5A). Similarly, no significant differences in expression of SERA5 protein were detected when comparing the chimWT and chimS596A clones by Western blot analysis (Fig. 5B–D). These results suggested that substitution of the SERA5 Ser596 residue in a manner predicted to ablate any catalytic activity did not result in detectable compensatory changes in expression of *SERA5* or other members of the *SERA* gene family, consistent with the notion that SERA5 function is not affected by the Ser596Ala substitution.

### Failure to truncate the *SERA5* gene, introduce a C-terminal epitope tag or introduce a Ser596Arg substitution confirms the essentiality of the *SERA5* gene

Encouraged by our ability to rapidly modify the *SERA5* gene by homologous recombination with the pHH1SERA5chimWT and pHH1SERA5chimS596A constructs, we decided to use a similar approach to re-examine the essentiality of the gene and its capacity to be further modified. For this, four new constructs were produced, all based on the previous constructs (Fig. 3B). Construct pHH1SERA5chimS596R was identical to pHH1SERA5chimWT except that it was designed to replace the putative catalytic Ser596 residue with a bulky, charged Arg residue. Integration of this construct was expected to modify the papain-like domain of SERA5 in a manner predicted to interfere with binding of substrate-like proteins to the active site-like cleft in the SERA5 papain-like domain (Supplementary Fig. S7). Construct pHH1SERA4chimTRUNC contained an in-frame STOP (TAA) codon within the recodonised synthetic sequence encoding the 3′ region of the *SERA5* ORF, such that integration should result in truncation of the target gene. The STOP codon in this construct replaced the codon for Glu650, which lies within the papain-like domain, so truncation at this point would be expected to entirely disrupt the structure of the domain. Construct pHH1SERA5chimHA3 was identical to pHH1SERA5chimWT except that at the 3′ end of the recodonised synthetic *SERA5* sequence, it included an in-frame 27 bp extension encoding 3 tandem haemagluttinin epitope tags (HA3), followed by a STOP codon. Integration of this construct was therefore predicted to fuse an HA3 tag to the extreme C-terminus of the SERA5 protein. Finally, construct pHH1SERA5chimΔP6mTAP was designed to replace the amino acid sequence of the P6 region that lies between site 2 and the P50C cleavage site (Fig. 1B) with a modified (‘mini’) tandem affinity-purification tag (mini TAP tag) based on the strategy of Rigaut *et al*. (1999), comprising an HA3 epitope tag sequence followed by a *Strep*-tag II sequence and a Tobacco Etch Virus (TEV) protease cleavage motif (Supplementary Fig. S8). This last construct was designed to achieve two functions; firstly, to address whether the bulk of the P6 segment could be deleted without modifying SERA5 function; and second, as a means of introducing an epitope and affinity-purification tag into SERA5 that might prove useful for future work investigating interactions between SERA5 and putative partner proteins. Parasites independently transfected with all four constructs were subjected to drug cycling as previously, and integration monitored by diagnostic PCR and/or IFA analysis as appropriate. In all cases, parallel transfection experiments were set up with the control pHH1SERA5chimWT construct to ensure the reproducibility of the transfection conditions and viability of the parasite cultures. The results of these experiments are summarised below and in Table 1.

**Table 1:** Summary of compiled transfection data

Construct name (pHH1SERA5chim)	Proportion of transfected cultures leading to integration^a^	Comments
WT	15/15	Integration PCR always positive by cycle 1
S596A	3/3	Integration PCR always positive by cycle 1
S596R	0/4	No integration by cycle 2 (2 expts) or cycle 6 (2 expts)
HA3	0/6	No integration by cycle 4 (2 expts) or cycle 6 (2 expts)
TRUNC	0/6	No integration by cycle 4 (2 expts) or cycle 6 (2 expts)
ΔP6mTAP	2/2	Nearly quantitative integration by cycle 3
WT plus TRUNC (mixture)	1/1	Nucleotide sequencing of integration-specific PR product at cycle 4 showed integration only of the WT construct

a Integration into the *SERA5* locus was detected by diagnostic PCR using PCR primers +27 and −11 (see Fig. 3).

In two independent transfection experiments (each performed in duplicate) with pHH1SERA5chimS596R, no integration could be detected by PCR even after six cycles of drug selection, whilst the pHH1SERA5chimWT control plasmid integrated rapidly as previously (Table 1). These results suggest that – in contrast to the Ser596Ala substitution – the substitution of Ser596 with a large charged residue is not tolerated. This indicates, in turn, that although SERA5 does not function as an essential enzyme in parasite asexual blood stages, the papain-like domain nonetheless has an essential function.

Transfection of parasites with both pHH1SERA5chimTRUNC and pHH1SERA5chimHA3 was performed in duplicate on three independent occasions, in all cases alongside duplicate cultures transfected with control construct pHH1SERA5chimWT. Integration of pHH1SERA5chimWT was again consistently detected by PCR following just a single drug cycle in all transfected cultures. However, no integration of either pHH1SERA5chimTRUNC or pHH1SERA5chimHA3 was detected even after drug cycle 6 in cultures of the first two independent transfection experiments, or by drug cycle 4 in cultures of the third independent transfection experiment (Table 1). In this third transfection experiment, as an additional control, a parasite culture was transfected with a mixture of plasmid DNA of pHH1SERA5chimWT and pHH1SERA5chimTRUNC in a 1:1 (w/w) ratio. In this case, again only pHH1SERA5chimWT integrated into the parasite genome as assessed by nucleotide sequencing of the products of the diagnostic integration PCR (Table 1). In summary, these results strongly indicate that truncation of the *SERA5* gene, or fusion of SERA5 to a C-terminal epitope tag, is lethal or severely deleterious to parasite growth.

**Figure 6:**
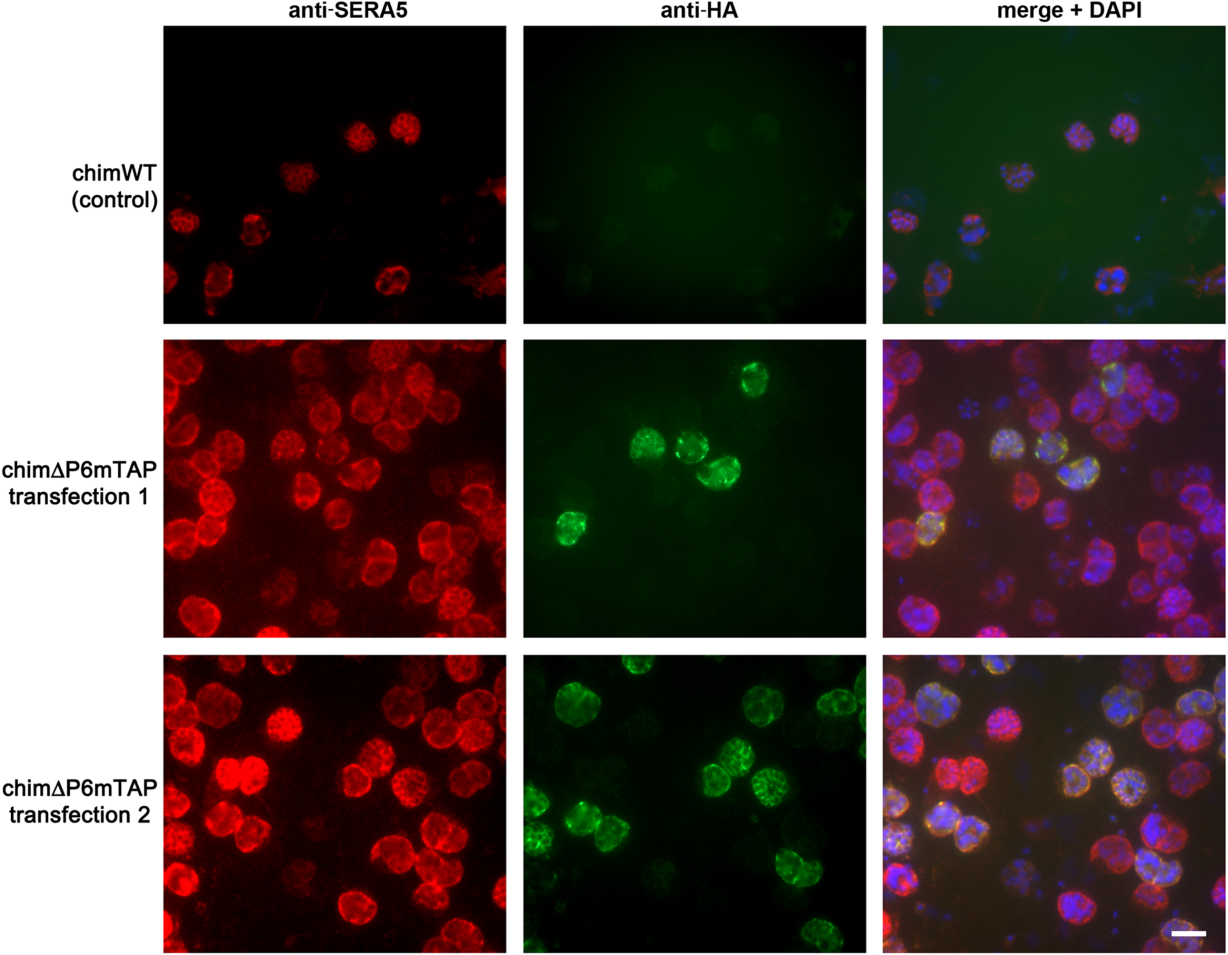
The P6 segment of SERA5 can be readily replaced with a tandem affinity-purification tag. IFA of uncloned drug-resistant parasite lines examined just one cycle of drug selection following transfection with control construct pHH1SERA5chimWT (top row), designed to simply reconstitute the wild-type SERA5 coding sequence, or with pHH1SERA5chimΔP6mTAP (middle and bottom rows), designed to introduce a mini TAP tag into the SERA5 coding sequence in place of the region encompassed by the P6 region (see Supplementary Fig. S8). Fixed parasites were probed with a polyclonal rabbit anti-SERA5 antibody (red) or with the anti-HA3 mAb 12CA5 (green). A significant proportion of the pHH1SERA5chimΔP6mTAP-transfected parasites exhibit an HA3-specific signal, indicating rapid integration of the transfection construct. Scale bar, 5 μm.

In contrast to the results of transfection with the above three constructs, duplicate transfection experiments with construct pHH1SERA5chimΔP6mTAP resulted in rapid integration by drug cycle 1 in all cases. Examination of uncloned drug-resistant cultures by IFA using anti-HA3 antibodies showed a strong positive signal in up to ∼ 20% of schizonts, which co-localised with the anti-SERA5 signal, indicating efficient integration of the input construct in the predicted manner (Fig. 6). These results indicate that the bulk of the P6 SERA5 segment is dispensable and can be replaced by unrelated sequence. Moreover, the ease and rapidity with which integrant parasite were established with this construct adds further weight to the conclusions from use of the other three constructs showing that truncation or C-terminal tagging of SERA5 or the substitution of Ser596 with an Arg residue is not tolerated, presumably because all three modifications disrupt an essential but non-enzymatic function of SERA5.

## Discussion

Interest from the malaria research community in SERA5 has spanned over three decades, provoked, in part, by the capacity of this highly abundant *P. falciparum* protein to induce potentially protective immune responses in animal malaria models and humans. Despite this and continued speculation over its potential role as a proteolytic enzyme, the function of SERA5 has remained enigmatic. Our new findings allow a number of unambiguous conclusions to be drawn while at the same time raising further questions about the biological role of SERA5.

Using mass spectrometric mapping of the P50 form of SERA5 that is released into culture supernatants following schizont rupture, we definitively identified the P50C site at which cleavage by protease X takes place to convert P56 to P50 (Fig. 1B). Cleavage occurs at 839DYYL↓KASP846, suggesting that protease X has a substrate specificity quite distinct from that of PfSUB1, which is unable to cleave on the C-terminal side of a Leu residue and generally requires acidic residues on the prime side of the scissile bond (Withers-Martinez *et al*., 2002; 2012). No attempt was made here to identify protease X, but our mapping of its target sequence may aid in that goal in future work, for example, through the development of selective substrates for protease X. More relevant to the present study, the mapping data confirmed that the P50 terminal processing fragment contains all the residues predicted to be encompassed within the papain-like domain of SERA5. Despite this, we were unable to detect hydrolytic activity associated with the parasite-derived protein or with recombinant SERA5 containing the wild-type Ser565 residue, either in intact form or following digestion *in vitro* with rPfSUB1. These results contrast with those of Hodder *et al*. (2003) and Kanodia *et al*. (2014) who demonstrated a chymotrypsin-like protease activity associated with a refolded, *E. coli*-derived recombinant form of the SERA5 papain-like domain. The reason for this discrepancy is unclear. However, we found that a recombinant protein containing a Cys substitution of Ser596 (rSERA5-C) displayed robust peptidase activity with the characteristics of a papain-like cysteine protease, including activation by DTT and sensitivity to E64. Whilst a definitive demonstration that a protein has (or lacks) peptidase activity can be difficult to achieve experimentally, our approach using a set of three homologous recombinant proteins that differ from each other at only a single residue and that were expressed and purified under identical conditions provides a high degree of confidence that the detected activity was mediated by rSERA5-C, rather than by a contaminating activity. This finding has profound implications. Earlier x-ray crystal structural analysis of the SERA5 papain-like domain by Hodder and colleagues raised questions about the capacity of the ‘active-site’ cleft of SERA5 to interact with peptide substrate, in main due to the non-canonical orientation of two residues (Gly639 and Ser640) situated along a strand that lines one side of the cleft, the presence of an Asp residue (Asp594) adjacent to Ser596 that limits access to the groove and the presence of a Tyr residue (Tyr735) that severely restricts the depth of the predicted S2 pocket (Hodder *et al*., 2009). Assuming that the S596C substitution does not significantly alter the overall structure of these other elements of the cleft, our finding that rSERA5-C displays peptidase activity *in vitro* conclusively proves that the SERA5 cleft – despite its unusual architecture – has retained a functional peptidase-like tertiary structure and can bind peptide substrates. This raises the possibility that the function of SERA5 in the parasite may involve substrate-like interactions with peptide or protein partners. Our inability to replace Ser596 in the parasite with an Arg residue supports that notion, since modelling indicates that this residue would extend out of the cleft into solvent, interfering with substrate-like interactions (Supplementary Fig. S7). Also supporting the model is evidence from others that peptide-based compounds designed to interact with the cleft of the SERA5 papain-like domain impact on parasite viability, although the target(s) and mechanism of action of those compounds in the parasite remain unclear (Fairlie *et al*., 2008; Alam and Chauhan, 2012; Kanodia *et al*., 2014). Arguing against the model is the fact that we consistently failed to detect any other proteins co-purifying with P50 isolated from parasite culture supernatants. However, it is conceivable that SERA5 engages in only low-affinity interactions with its putative partners and/or that these interactions are reversed rapidly following egress, perhaps as a result of proteolytic processing of SERA5 by PfSUB1 and/or protein X. It is worth noting that structural changes to the active-site cleft of papain have been observed upon inhibitor binding (Drenth *et al*., 1976), providing some optimism that the SERA5 cleft may be capable of accommodating (by induced fit) more extended peptide partners than predicted from the apparently restricted nature observed in the X-ray crystal structure. Further exploration of the substrate specificity of our recombinant rSERA5-C peptidase may provide useful insights into the structural characteristics required for binding in the cleft.

We were initially surprised by our inability to obtain integrant parasites using transfection construct pHH1SERA5chimHA3, suggesting as it does that fusion of an HA3 epitope tag to the C-terminus of SERA5 is deleterious. However, previous work in this laboratory on the other essential *P. falciparum* SERA family member SERA6 reached a similar conclusion; although the *SERA6* gene could be targeted by integration constructs (Ruecker *et al*., 2012), we were unable to incorporate a C-terminal HA3 tag (M. Shea. A. Ruecker and M. Blackman, unpublished). We speculate that this may be due to the presence of a hydrophobic six-residue segment, including a pair of completely conserved Cys residues, right at the C-terminus of all known *Plasmodium* SERA family members (Arisue *et al*., 2011). If this segment folds into the core of the protein and/or if the Cys residues are involved in intramolecular disulphide bonds (e.g. with the P47 N-terminal domain; see Fig. 1B), the presence of an adjacent tag may fatally interfere with protein folding. In contrast, insertion by homologous recombination of a mini TAP tag into the SERA5 sequence in place of the P6 segment was rapidly achieved. This allowed us to confirm expression of the chimeric protein by detection of the tagged SERA5. Our demonstration that the P6 region is dispensable for SERA5 function is of interest in the light of a previous suggestion that this segment acts to regulate SERA5 function in the parasite (Kanodia *et al*., 2014). Our data prove that this is not an essential regulatory role.

In spite of the peptidase activity displayed by rSERA5-C *in vitro*, the most important conclusion arising from our study is that wild-type SERA5 does not perform an essential enzymatic role in the parasite, since parasites possessing a S596A substitution displayed *in vitro* growth rates and morphology indistinguishable from wild-type parasites. The fact that in parallel experiments – using similar constructs with identical targeting regions – we could not truncate the *SERA5* gene, modify its 3′ end or integrate the S596R mutation provides compelling confirmation that (a) the S596A mutation is not a loss-of-function mutation; and (b) that the function of SERA5 in blood stages cannot be complemented by any other member(s) of the SERA family. This is also borne out by our inability to detect any upregulation of other *SERA* genes in the S596A mutant. So what role could SERA5 play? We actually believe it unsurprising that wild-type SERA5 does not exhibit detectable peptidase activity, since experimental replacement with Ser of the nucleophilic Cys in papain, cathepsin L, cathepsin S and the related clan CA enzyme μ-calpain abolishes enzyme activity (Clark and Lowe, 1978; Coulombe *et al*., 1996; Turkenburg *et al*., 2002; Hata *et al*., 2013), and numerous examples of active-site Cys-to-Ser mutations of other cysteine proteases including the clan C1B protease bleomycin hydrolase (O’Farrell *et al*., 1999) and several clan CD caspases (e.g. Van Criekinge *et al*., 1996) have shown that this mutation invariably results in loss of catalytic activity. Indeed, we are not aware of a single example in which substitution with Ser of the active-site Cys nucleophile in a cysteine peptidase scaffold leads to retention of significant catalytic activity. Naturally occurring papain-like proteins with Ser at the position of the catalytic Cys are well documented and include a class of silica-condensing and hydrolytic enzymes termed the silicateins (for a recent review, see Muller *et al*., 2013); but even in the case of these enzymes, replacement of the catalytic Ser with Ala abolishes catalysis (Cha *et al*., 1999). We therefore believe that the simplest interpretation of our data is that SERA5 does not perform a catalytic role in the parasite at all. Inactive enzyme homologues, or pseudoenzymes, are widespread across evolution (Bartlett *et al*., 2003; Pils and Schultz, 2004). For the great majority of these their function is unknown, but those few cases where function has been ascribed suggest that pseudoenzymes may often be involved in regulation of their active enzyme counterparts (see Adrain and Freeman, 2012 and Reese and Boyle, 2012, for recent reviews). This can be mediated through a number of mechanisms including allosteric activation of the active homologues by complex formation (Willert *et al*., 2007) and inhibition of the active homologues by blocking access to substrate (Devedjiev *et al*., 1997). Pseudoenzymes can also regulate the activity of heterologous enzymes, as in the case of iRhoms, a subset of the rhomboid family of intramembrane serine proteases that lack important catalytic residues (recently reviewed by Bergbold and Lemberg, 2013); these can act as important regulators of the trafficking and fate of rhomboid substrates, rhomboids themselves and even proteolytic enzymes of other mechanistic classes (Zettl *et al*., 2011; Adrain *et al*., 2012; Christova *et al*., 2013). Whether inactive variants of cysteine proteases can function in an analogous manner is less clear, but recent studies have suggested that this is possible; for example, a proteolytically inactive Cys-to-Ser variant of a *Trypanosoma brucei* metacaspase (clan CD) was found to act as a virulence factor and play an important role in bloodstream parasite viability, implying a regulatory role (Proto *et al*., 2011). Could SERA5 have an analogous function? In all *Plasmodium* species examined, the SERA family includes both ‘Ser-type’ genes such as SERA5 [also referred to as group IV genes (Arisue *et al*., 2007)], and ‘Cys-type’ genes which more closely resemble canonical papain-like proteases in that they possess a Cys in the active-site position. Phylogenetic analyses have suggested that the Ser-type genes evolved by gene duplication from an ancestral Cys-type gene (Hodder *et al*., 2003; Arisue *et al*., 2007;,^2011^). Together with experimental data indicating that disruption of the *SERA4* gene results in an upregulation of *SERA5* expression, this has led to the suggestion that all the Ser-type family members have a common function (Bourgon *et al*., 2004; McCoubrie *et al*., 2007). If, as our new data suggest, that function involves interacting with client molecules in a substrate-like manner, there may be selective pressure to maintain a protease-like architecture within the cleft, whilst avoiding actual proteolytic activity; this could explain the common possession of a Ser replacing the canonical Cys and the minimal number of other substitutions observed around the ‘catalytic’ cleft (Arisue *et al*., 2011). We recently showed that the other essential PV-located *P. falciparum* blood stage SERA family member, SERA6, which is a Cys-type SERA, may be an essential protease in blood stages (Ruecker *et al*., 2012). Like SERA5, SERA6 is implicated in the protease-mediated pathway leading to egress. We speculate that SERA5 (and possibly all the other Ser-family members) acts to regulate the function of SERA6, perhaps by controlling the timing of access of SERA6 to its substrate(s). This regulatory function may be dispensable in some *Plasmodium* species since disruption of both of the two Ser-type *SERA* genes in the rodent malaria species *P. berghei* had no phenotypic consequences, the knockout parasites progressing normally through their entire life cycle (Putrianti *et al*., 2010). This result might reflect important differences between *Plasmodium* species in their mode of blood stage egress; whereas in *P. falciparum*, as well as the zoonotic pathogen *P. knowlesi* (Dvorak *et al*., 1975) and the rodent species *P. yoelii* (Yahata *et al*., 2012), schizont rupture occurs efficiently in static cultures, egress in *P. berghei* appears to require the shear stresses imposed by schizont flow through the microvasculature (Janse *et al*., 2006). Given the apparently essential nature of SERA5 in *P. falciparum*, much could be gleaned of its function through the application of a conditional gene disruption approach such as the recently described DiCre system (Collins *et al*., 2013b), and work towards that goal is underway. We predict that further studies on the role of the SERA protein family will produce significant mechanistic insights into the regulation of egress in the malaria parasite.

## Experimental procedures

### Reagents, synthetic *SERA5* gene and antibodies

Activity-based probes FP-biotin (Liu *et al*., 1999) and JCP104 (Arastu-Kapur *et al*., 2008) were kind gifts of Matthew Bogyo (Stanford University, CA, USA). Tunicamycin, E64 and PMSF were from Sigma. Fluorogenic substrates were obtained from Sigma or Calbiochem. The antifolate drug WR99210 was from Jacobus Pharmaceuticals (NJ, USA). A previously described (Collins *et al*., 2013b), synthetic *P. falciparum* 3D7 *SERA5* gene (*SERA5_synth_*), lacking introns and with codon usage optimised for *E. coli*, was assembled in plasmid pMKrecodSERA5. ClaI and SalI restriction sites within the recodonised *SERA5* ORF at positions 1222–1227 and 2464–2469 respectively were used frequently in the cloning procedures. Monoclonal antibody (mAb) 89.1, which recognises the major merozoite surface protein MSP1, has been described previously (Holder and Freeman, 1982) as have the human anti-MSP1 mAb X509 (Blackman *et al*., 1991), the anti-SERA5 mAb 24C6.1F1 (Delplace *et al*., 1985; a kind gift from Jean-François Dubremetz, University Montpellier 2, France), and a rabbit antibody to *P. falciparum* AMA1 (Collins *et al*., 2009).

### Culture, transfection, cloning and *P. falciparum* growth assays

Asexual blood stages of *P. falciparum* clone 3D7 were cultured in an atmosphere of 90% nitrogen, 5% carbon dioxide and 5% oxygen at 37°C in RPMI 1640 medium containing Albumax (Invitrogen) supplemented with 2 mM L-glutamine, and synchronised using standard procedures (Blackman, 1994; Harris *et al*., 2005). Parasite developmental stage and viability were routinely assessed by microscopic examination of Giemsa-stained thin blood films. For transfection, ring-stage parasites (5% parasitaemia in 200 μl blood) were electroporated with plasmid DNA (70–100 μg per transfection) as described previously (Harris *et al*., 2005). Growth medium was replaced ∼ 24 h post transfection with fresh medium containing 2.5 nM WR99210. Once parasites displayed robust growth (2–3 weeks post-transfection), they were subjected to repeated cycles of culture for 3 weeks without drug followed by culturing with drug (drug cycling) to select for parasites in which integration into the genome had taken place (Harris *et al*., 2005). Clones were obtained by limiting dilution, plating a calculated 0.1 parasite per well in 96-well round-bottomed microplates. Once established, all transgenic clones were cultured routinely in medium containing WR99210.

For growth assays, synchronous mature schizonts of two chimWT parasite clones and two chimS596A clones (each pair originating from two independent transfection experiments), as well as the pHH1SERA5chimWT-transfected but non-integrant clone i, were enriched by centrifugation on a Percoll cushion (GE Healthcare), added to fresh erythrocytes and allowed to undergo invasion for 30 min, then depleted of residual schizonts by repeated centrifugation over Percoll cushions followed by sorbitol treatment. The resulting cultures, containing only young ring-stage forms, were cultured for 40 h before assessing parasitaemia by fluorescence-activated cell sorter (FACS) analysis (see discussion later). All cultures were then adjusted to 0.5% parasitaemia, 2% haematocrit in triplicate wells of six-well plates and incubated in a humid chamber. After each (or every second) growth cycle, parasitaemia was determined by FACS and each culture was adjusted back to a 0.5% parasitaemia in fresh six-well plates by dilution into fresh erythrocytes at 2% haematocrit in fresh medium. The dilution factors required to achieve this gave a measure of the relative growth rates of the clones. Microscopic examination of Giemsa-stained thin films was routinely used as an additional confirmation of parasite viability.

For parasitaemia measurements by FACS, parasites recovered at various time points were stained with the fluorescent vital stain hydroethidine (HE) (Bergmann-Leitner *et al*., 2006). Stock solutions of HE (10 mg ml^−1^ in dimethyl sulfoxide (DMSO)) were freshly diluted 1:200 into warm phosphate buffered saline (PBS), then 500 μl of the dilution added to 50 μl of parasite culture and incubated for 20 min at 37°C. The samples were then diluted into 1 ml PBS and stored in the dark on ice for up to 2 h to stop HE uptake by viable parasites. As negative controls, uninfected erythrocytes in culture medium were stained and processed in the same way. Parasitaemia was calculated using the FACSCalibur flow cytometer (Becton Dickson) as described previously (Moss *et al*., 2012). Briefly, cultures to be analysed were initially screened using forward and side scatter parameters and gated for erythrocytes. From this gated population, the proportion of HE-stained cells in 100 000 cells was determined using the FL2 detector (585/42 nm).

### Purification of parasite-derived SERA5 P50 and mapping the protease X (P50C) cleavage site

Parasite-derived P50 was purified from supernatants of synchronous mature 3D7 schizonts that had been allowed to undergo egress and invasion overnight in RPMI 1640 medium lacking Albumax. Supernatants were clarified by centrifugation and filtration, supplemented with 1 M Tris–HCl pH 8.2 to 50 mM Tris–HCl, then applied directly to a HiPrep 16/10 QXL anion exchange column (GE Healthcare). The column was washed with 50 mM Tris–HCl, 150 mM NaCl pH 8.2, then bound proteins eluted with a 500 ml gradient of 150–500 mM NaCl in the same buffer. Peak fractions were pooled, concentrated and further chromatographed on a HiLoad 26/60 Superdex 200 prep-grade column (GE Healthcare) equilibrated in 25 mM (4-(2-hydroxyethyl)-1-piperazineethanesulfonic acid (HEPES), 150 mM NaCl, pH 7.0. Purified P50 was stored in aliquots at −70°C. Electrophoretic transfer of purified protein (∼ 10 μg) to PVDF membrane for N-terminal sequencing (performed by by the Protein and Nucleic Acid facility at the Department of Biochemistry, University of Cambridge, UK) and electrospray mass spectrometry of the intact protein using a Bruker microTOFQ was as described previously (Ruecker *et al*., 2012).

For C-terminal analysis by limited carboxypeptidase Y digestion, purified P50 was adjusted to a concentration of ∼ 500 μg ml^−1^. From a sample of 100 μl, a ‘START’ aliquot (10 μl) was removed and added to a fresh tube containing 2 μl 5% (v/v) trifluoroacetic acid, then frozen on solid CO_2_. To the remaining P50, protein was added 0.8 μg (2 μl) freshly made-up sequencing grade carboxypeptidase Y (Roche), and the mixture was incubated at 37°C with further aliquots of 10 μl being withdrawn at intervals of 5, 10, 20, 30, 60 min and 3 h, in all cases adding them immediately to TFA as earlier to stop the reaction. All samples were then analysed by electrospray mass spectrometry as earlier.

### Transfection constructs for targeted modification of the *P. falciparum* *SERA5* gene

All constructs for integration into the parasite genome by single crossover homologous recombination were based on the previously described construct pHH1SERA5chimWT (Collins *et al*., 2013b). Construct pHH1SERA5chimS596A was obtained by megaprimer extension-based site-directed mutagenesis as described previously (Stallmach and Gloor, 2008). Megaprimers were obtained by PCR amplification from pMKrecodSERA5, using the oligonucleotide primer pairs +S5Seq1021 with −S5_StoA_BstUI, and +S5_StoA_BstUI with −S5stopXhoI respectively (see Supplementary Table S2 for a list of all primers used in this work). Primers −S5_StoA_BstUI and +S5_StoA_BstUI introduce a BstUI site overlapping the codon for Ala596. The resulting PCR products (‘megaprimers’) were then used as template in an overlap extension PCR with external primers +S5Seq1021 and −S5stopXhoI. The resulting amplicon was digested with ClaI and SalI and cloned into ClaI/SalI-digested pHH1SERA5chimWT, replacing the middle segment of the recodonised ORF that encodes the wild-type Ser596. Constructs pHH1SERA4chimTRUNC and pHH1SERA5chimS596R were generated in a similar manner using internal primers −S5XbaIKO with +S5XbaIKO (which contain an XbaI site overlapping with an in-frame premature stop codon introduced downstream of the Ile649 codon) and −S5StoR_MluI with +S5StoR_MluI (which introduces a MluI site overlapping the Arg596 codon) respectively. Construct pHH1SERA5chimHA3 was obtained by replacing the segment between the pHH1SERA5chimWT SalI and XhoI sites with the corresponding segment of pMKrecodSERA5 which does not bear a stop codon before the XhoI site, resulting in a continuous reading frame into the HA3 tag in the final pHH1SERA5chimHA3 construct. Construct pHH1SERA5chimΔP6mTAP was obtained by overlap extension PCRs (to introduce the mini TAP tag) using oligonucleotide pairs +S5Seq2021 with −1longHS and +2longHS with −29_S5stopXhoI respectively, using pHH1SERA5chimWT as template. Read-through PCR using the PCR products as templates was performed with external primers +S5Seq2021 and −29_S5stopXhoI. The resulting PCR product was inserted into pHH1SERA5chimWT via the SalI and XhoI sites, leading to construct pHH1SERA5chimΔP6mTAP.

### Recombinant expression of SERA5 variants

For recombinant expression of rSERA5wt and mutants thereof, the honey bee melittin secretion signal was first amplified by PCR from plasmid pMIB/V5-His (Invitrogen) using the oligonucleotide pair +BamHImelit plus −melitSERA5. This primer extends the melittin encoding sequence in frame by bases complementary to the recodonised *SERA5* gene downstream of the endogenous SERA5 secretory peptide signal. The resulting PCR product was mixed with pMKrecodSERA5 and overlap extension PCR was performed using primer pair +BamHImelit with −S5ENDHindIII. The product, encoding SERA5 with a melittin secretion signal instead of the endogenous sequence, was inserted into the BamHI/HindIII sites of pFastBac1 (Invitrogen) resulting in construct pFastBac1SERA5-WT. Mutant constructs pFastBac1SERA5-S596C and pFastBac1SERA5-S596A were generated by megaprimer extension-based site-directed mutagenesis as earlier with internal primers −S5_StoC plus +S5_StoC and −S5_StoA_BstUI with +S5_StoA_BstUI respectively.

Recombinant bacmids were produced from pFastBac1SERA5-WT, pFastBac1SERA5-S596C and pFastBac1SERA5-S596A using the Bac-to-Bac^TM^ Baculovirus Expression System (Invitrogen), following the manufacturer’s protocols. Viral plaque assays were conducted to determine the viral titre of P3 stocks which were then used to infect High Five^TM^ insect cells (Invitrogen) at a multiplicity of infection of ∼ 1 in Sf-900 II SFM medium with or without the addition of tunicamycin (0.1 μg ml^−1^) to prevent protein N-glycosylation. Accumulation of secreted recombinant protein in the medium was monitored by Coomassie-stained sodium dodecyl sulfate–polyacrylamide gel electrophoresis (SDS–PAGE). After ∼ 3 days, culture supernatants were harvested, clarified by centrifugation and either frozen or directly submitted to the purification protocol.

To purify rSERA5wt, clarified insect cell culture supernatants were loaded onto a HiPrep 16/10 QXL anion exchange column (GE Healthcare) at a flow rate of 5 ml min^−1^. The column was washed with 20 mM Bis–Tris, 150 mM NaCl pH 6.5 and bound protein eluted at 1 ml min^−1^ using a gradient of 150–500 mM NaCl in the same buffer. The column was extensively cleaned with 0.5 M NaOH before being re-used for rSERA5-A and rSERA5-C. Eluate fractions containing recombinant protein were pooled, concentrated using a CentriconPlus-70 30 kDa molecular mass cut-off ultrafiltration device, then chromatographed on a HiLoad 26/60 Superdex 200 prep-grade column (GE Healthcare) equilibrated in 20 mM HEPES, 150 mM NaCl, pH 7.4. The identity of the purified recombinant SERA5 proteins was confirmed by Western blot and/or N-terminal sequencing, and purity and monodispersity were assessed by Coomassie and silver staining as well as dynamic light scattering. The purified proteins were divided into aliquots and stored at −80°C. Yields were ∼ 20 mg of purified recombinant SERA5 per liter of High Five^TM^ cell supernatant. Purified rSERA5wt was used to immunise a rabbit (performed by Harlan Laboratories, UK) to produce a polyclonal anti-SERA5 rabbit antiserum.

### Zymogram assays for protease activity

To assess proteolytic activity of purified parasite-derived P50 or recombinant SERA5 variants rSERA5wt, rSERA5-A and rSERA5-C, intact or PfSUB1-digested test proteins were incubated in non-reducing SDS loading buffer for 15 min at room temperature before being subjected to SDS–PAGE (20–100 ng of protein per well) in 10% gels containing 1 mg ml^−1^ copolymerised protein substrates (heat-denatured porcine gelatin or BSA). Following electrophoresis, gels were incubated in 2.5% (v/v) Triton X-100 in deionised water twice for 15 min with agitation at room temperature. Gels were then equilibrated in one of five different developing buffers (25 mM Tris–HCl pH 7.4, 12 mM CaCl_2_; 25 mM HEPES pH 7.0, 150 mM NaCl_2_, 1 mM DTT; 50 mM phosphate buffer pH 6.8, 1 mM EDTA, 1 mM DTT; 100 mM sodium acetate pH 6.0, 1 mM DTT; or 100 mM sodium acetate pH 6.0, 5 mM CaCl_2_) for 30 min at room temperature before being further incubated at 37°C for 23 h in a fresh change of the same developing buffer. Gels were finally stained with 0.5% (w/v) Coomassie Blue R-250 for 30 min and destained until areas of protease activity appeared as clear bands against a blue background. Chymotrypsin was used as a control protease in these assays.

### Peptidase activity assays using unlabelled and fluorogenic peptide substrates

A range of unlabelled and fluorogenic peptide substrates (listed in Supplementary Table S1 together with assay details and buffer conditions) were used to screen purified parasite P50 and recombinant proteins rSERA5wt, rSERA5-A and rSERA5-C for peptidase activity. For detailed inhibitor profiling and kinetic assays using the rSERA5-C substrate Abz-LAQAVRSSSR-Dpa, substrate was diluted from a 5 mM stock in DMSO into optimised buffer (25 mM Bis–Tris pH 6.5, 1 mM EDTA) containing no additions or a range of concentrations of DTT, E64 or PMSF, before being dispensed in 100 μl aliquots into white 96-well microplates (FluoroNunc, NUNC). Wells were then supplemented with ∼ 1 μg (2–5 μl) rSERA5-C or control protein (P50, rSERA5wt or rSERA5-A). Substrate hydrolysis (increase in fluorescence) was then continuously monitored at 5 min intervals at room temperature using a Cary Eclipse fluorescence spectrophotometer (Varian) equipped with a 96-well microplate reader accessory (λ_ex_ 320 nm, λ_em_ 420 nm), as previously described (Blackman *et al*., 2002). The machine was blanked on wells containing substrate only. Initial rates of hydrolysis were calculated under conditions in which < 10% of substrate had been hydrolysed, as determined by comparison with wells in which hydrolysis had been allowed to go to completion.

### Diagnostic PCR and Southern blot analysis

Genomic DNA was isolated from cultured parasites using the DNeasy blood and tissue kit (Qiagen). Diagnostic PCR to screen for integration of transfection constructs (integration PCR) was performed as previously described (Collins *et al*., 2013b) using forward primer +27, designed to hybridise to the second exon of the *SERA5* gene, with reverse primer −11 which anneals in the recodonised region of the chimeric *SERA5* gene (see Fig. 3 for orientation). The presence of the unmodified endogenous *SERA5* gene was detected using the same forward primer together with reverse primer −25, which anneals in the equivalent region as reverse primer −11 but hybridises selectively to endogenous *SERA5* gene sequence. PCR was conducted using 2X KAPA2G Fast HotStart ReadyMix (KAPABIOSYSTEMS) with 2 min initial denaturation at 95°C followed by 35 cycles of 15 s at 95°C followed by 15 s of annealing at 60°C and 10 s extension at 72°C, concluded by a final extension step of 3 min at 72°C. For Southern blot, genomic DNA from parental clone 3D7 parasites or cloned transgenic parasites was digested with EcoRV, fractionated by agarose gel electrophoresis on 0.7% agarose gels and transferred to Hybond-N nylon membranes (GE Healthcare). Probe template was generated by PCR using forward primer b (annealing to the third exon of the *SERA5* gene) with reverse primer −S5endogClaI, purified by agarose gel electrophoresis followed by a QIAquick gel extraction (Qiagen) step and then used to generate ^32^[P]-labelled probe using the Prime-It II Random Primer labeling Kit (Stratagene) according to the manufacturer’s recommendations. The probe was purified using ProbeQuant^TM^ G50 Micro Columns (Amersham Biosciences), boiled and used for hybridisation overnight at 62°C in SSC.

### Quantitative reverse transcription real-time PCR (RT-qPCR)

For cDNA preparation, cultures of four cloned parasite lines (two chimWT clones and two chimS596A clones) as well as the parental 3D7 clone were synchronised and used for RNA preparation at mature schizont stage. Total RNA was isolated with an RNeasy Mini kit (QIAGEN) according to the manufacturer’s protocol. To remove contaminating DNA, 7 μg of total RNA from each clone was treated with TURBO™ DNase and DNase Inactivation Reagent (Ambion) according to the manufacturer’s protocol. A sample (3 μg) of each treated total RNA was submitted to a cDNA synthesis reaction either in the presence or absence (negative control) of SuperScript® II Reverse Transcriptase (RT, Invitrogen), using random primers and in the presence of RNasin according to the manufacturer’s protocol. Oligonucleotide primers for the RT-qPCR reactions (purchased in desalted form from Sigma) were designed for all nine *P. falciparum* 3D7 *SERA* family members, as well as the *P. falciparum β-tubulin*, *AMA1* and *MSP1* genes. Forward and reverse oligonucleotide primers (all with names beginning with ‘Q’ in Supplementary Table S2) were designed using Beacon Designer™ free edition (PREMIER Biosoft) to be 21–33 bp in length, with annealing temperatures close to 60°C, guanine–cytosine (GC) content around 30%, hairpin and hetero-/homodimer minimisation, and predicted to produce cDNA-derived amplicons of between 102 and 154 bp in length. In the case of the *SERA* genes, primers were designed to span approximately equivalent regions of the gene transcripts that encompass two exons.

RT-qPCR was performed using the Applied Biosystems 7500 Fast Real-Time PCR System and results were analysed with 7500 Software v2.0.5 (Applied Biosystems). Reactions were carried out in 96-well microplates, using 2× QuantiFast SYBR Green PCR Master Mix (QIAGEN) according to the manufacturer’s protocol. RT-qPCR conditions used were as follows: 5 min PCR activation at 95°C followed by 40 rounds of two-step cycling with 10 s denaturation at 95°C and 30 s combined annealing/extension at 60°C. Initially, RTq-PCR reactions were performed using a series of 4fourfold serial dilutions of a mixture containing 20% of each of the five cDNA preparations under analysis. The resulting data were used to generate initial reference curves to confirm efficiency and dynamic range compliance of all primer pairs under the chosen RT-qPCR conditions. A mix of the non-RT reactions at the highest concentration available was used to confirm the absence of DNA template. The product of one RT-qPCR from one of the dilution series with the primers designed to amplify *SERA8* was confirmed by nucleotide sequencing. Then, for each cDNA preparation, all 12 genes under analysis were assayed in triplicate, including no RT and no template controls on the same plate. Maximally, three samples were excluded per 96 well plate after melting curve analysis (never more than one of a given triplicate sample set). Standard deviation values between technical replicates were usually 0.1–0.2 threshold cycles (C_T_). The *β-tubulin* gene was used as the reference gene in each cDNA sample set and its C_T_ number was subtracted from the C_T_ of every other gene to obtain the corresponding ΔC_T_ value. RT-qPCR data generated with the cDNA of the parental 3D7 line was used as calibrator and the ΔC_T_ values of all its analysed genes were subtracted from the corresponding ΔC_T_ values of all the other samples to obtain the ΔΔC_T_ values for each gene.

### Western blot and indirect IFA

Western blot analysis using rabbit anti-SERA5, rabbit anti-AMA1 or the anti-MSP1 mAb X509 was performed as described previously (Jean *et al*., 2003). For IFA, thin blood films prepared from synchronous *P. falciparum* cultures enriched in mature schizonts were air dried, fixed with 4% (w/v) paraformaldehyde in PBS for 20 min and incubated in blocking buffer [10% (v/v) fetal calf serum, 3% (w/v) BSA, 0.5% (w/v) glycine in PBS] for 30 min at room temperature. Slides were probed with polyclonal rabbit anti-SERA5 (diluted 1:6000), anti-MSP1 mAb 89.1 ascites (diluted 1:1000) or purified anti-HA3 mAb 12CA5 (0.1 μg ml^−1^), all diluted in blocking buffer containing 0.1% (w/v) saponin. The slides were washed in PBS 0.05% (v/v) Tween 20 (PBS-Tween) then bound primary antibodies detected using Alexa Fluor 488 or Alexa Fluor 594-conjugated anti-mouse or anti-rabbit secondary antibodies (Invitrogen) diluted 1:2000 in blocking buffer containing 0.1% (w/v) saponin. Samples were washed in PBS-Tween, stained with 4,6-diamidino-2-phenylindole (DAPI), mounted in Citifluor (Citifluor Ltd., Canterbury, UK), and images were captured using AxioVision 3.1 software on an Axioplan 2 Imaging system (Zeiss) using a Plan-APOCHROMAT 1006/1.4 oil immersion objective.
