# Supplementary material for: Plasmodium falciparum SERA5 plays a non-enzymatic role in the malarial asexual blood-stage lifecycle
Source: Mol Microbiol. 2015 Feb 11;96(2):368–87. doi: 10.1111/mmi.12941 (PMC4671257; doi:10.1111/mmi.12941)
Supplement: Supplementary file 1 [file mmi0096-0368-sd1.pdf]

Supporting information

Plasmodium falciparum SERA5 plays a non-enzymatic role in the malarial asexual blood-stage lifecycle

Robert Stallmach[1], Manoli Kavishwar[1], Chrislaine Withers-Martinez[1], Fiona Hackett[1], Christine R. Collins[1], Steven A. Howell[2], Sharon Yeoh[1], Ellen Knuepfer[1], Avshalom J. Atid[1], Anthony A. Holder[1] and Michael J. Blackman[1]*

[1]Division of Parasitology, and [2]Division of Molecular Structure, MRC National Institute for Medical Research, London NW7 1AA, UK.

*For correspondence: E-mail mblackm@nimr.mrc.ac.uk; Tel. (+44) 208 816 2127; Fax (+44) 208 816 2730

Running title: A non-enzymatic essential function for SERA5

Keywords: malaria/*Plasmodium falciparum*/egress/SERA5/protease

Fig. S1. Primary sequence of *P. falciparum* SERA5 and positions of proteolytic processing sites.

Shown is the complete primary sequence of the *P. falciparum* 3D7 SERA5 gene product (PlasmoDB ID: PF3D7_0207600). Positions of cleavage by PfSUB1 are indicated in red (site 2 and site 3, as well as the allele-specific site 1; (Yeoh *et al.*, 2007, Debrabant *et al.*, 1992, Li *et al.*, 2002)). The position of cleavage by protease X (Leu842), defined in this study as the P50C cleavage site, is also indicated. The sequence encompassed by the terminal P50 processing fragment is shaded in pink and the papain-like domain Ser558-Lys832 is underlined, with the predicted catalytic triad residues Ser596, His762 and Asn787 shaded in yellow and marked with asterisks. The N-terminal secretory signal peptide is shaded in grey.

```
MKSYISLFFILCVIFNKNVIKCTGESQTGNTGGGQAGNTGGDQAGSTGGSPQGSTGASPQ 60

GSTGASPQGSTGASQPGSSEPSNPVSSGHSVSTVSVSQTSTSSEKQDTIQVKSALLKDYM 120

GLKVTGPCNENFIMFLVPHIYIDVDTEDTNIELRTTLKKTNNAISFESNSGSLEKKKYVK 180
                    ↓site 3
LPSNGTTGEQGSSTGTVRGDTEPISDSSSSSSSSSSSSSSSSSSSSSSSSSSSESLPANG 240

PDSPTVKPPRNLQNICETGKNFKLVVYIKENTLILKWKVYGETKDTTENNKVDVRKYLIN 300

EKETPFTNILIHAYKEHNGTNLIESKNYAIGSDIPEKCDTLASNCFLSGNFNIEKCFQCA 360
                              ↓site 1
LLVEKENKNDVCYKYLSEDIVSKFKEIKAETEDDDEDDYTEYKLTESIDNILVKMFKTNE 420

NNDKSELIKLEEVDDSLKLELMNYCSLLKDVDTTGTLDNYGMGNEMDIFNNLKRLLIYHS 480

EENINTLKNKFRNAAVCLKNVDDWIVNKRGLVLPELNYDLEYFNEHLYNDKNSPEDKDNK 540
                                                        *
GKGVVHVDTTLEKEDTLSYDNSDNMFCNKEYCNRLKDENNCISNLQVEDQGNCDTSWIFA 600

SKYHLETIRCMKGYEPTKISALYVANCYKGEHKDRCDEGSSPMEFLQIIEDYGFLPAESN 660

YPYNYVKVGEQCPKVEDHWMNLWDNGKILHNKNEPNSLDGKGYTAYESERFHDNMDAFVK 720
                                            *
IIKTEVMNKGSVIAYIKAENVMGYEFSGKKVQNLCGDDTADHAVNIVGYGNYVNSEGEKK 780
      *
SYWIVRNSWGPYWGDEGYFKVDMYGPTHCHFNFIHSVVIFNVDLPMNNKTTKKESKIYDY 840
  ↓P50C                                        ↓site 2
YLKASPEFYHNLYFKNFNVGKKNLFSEKEDNENNKKLGNNYIIFGQDTAGSGQSGKESNT 900

ALESAGTSNEVSERVHVYHILKHIKDGKIRMGMRKYIDTQDVNKKHSCTRSYAFNPENYE 960

KCVNLCNVNWKTCEEKTSPGLCLSKLDTNNECYFCYV                        997
```

Fig. S2. Degradation of purified rSERA5-C is not the result of a contaminating protease activity.

Purified recombinant proteins rSERA5-C and rSERA5-A (in 25 mM HEPES pH 7.4, 150 mM NaCl) were incubated either alone or as a mixture containing approximately equivalent amounts (~2 μg) of the purified proteins, and sampled either immediately (start) or after 4 h at 37°C for analysis by SDS PAGE and Coomassie staining. The rSERA5-C underwent extensive degradation over the period of the incubation (compare the 1st and 4th tracks from the left), whereas rSERA5-A was essentially stable (compare the 3rd and 6th tracks from the left). The SERA5-A also remained stable in the mixed samples (compare the 2nd and 5th tracks), indicating that the rSERA5-C preparation does not contain a protease that is capable of degrading rSERA5-A *in trans*. Note that the batches of both proteins used for this experiment displayed more degradation immediately following purification than the (different) batches used for the experiment presented in Fig. 2B of the main manuscript.

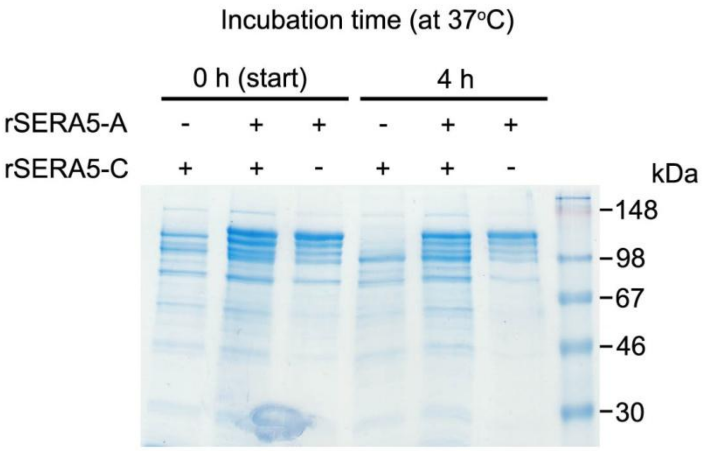

Fig. S3. Cleavage of TACE substrate IV by rSERA5-C takes place predominantly at the Arg-Ser bond.

A. Reversed-phase (RP)-HPLC elution chromatograms of mock-digested and rSERA5-C-digested TACE substrate IV (Abz-LAQAVRSSSR-Dpa). Digestions were carried out for 4 h at 37°C in 100 μl 25 mM Bis-Tris buffer pH 6.5 containing 1 mM EDTA, 10 mM DTT and 0.05% (w/v) Tween 20, using a substrate concentration of 10 μM and either no protein additions (mock digested) or 1 μg purified rSERA5-C. Digests were then fractionated by RP-HPLC chromatography on a Vydac 4.6 mm × 25 cm C_{18} reversed-phase column, eluted at 1 ml min^{-1} with a 18-54% (v/v) gradient of acetonitrile in 0.1% TFA over 15 min, with continuous monitoring of eluted components using a Jasco FP-920 fluorescence detector (λ_{ex} 320 nm, λ_{em} 420 nm). RP-HPLC-analysis of undigested substrate, or substrate incubated under similar conditions with purified rSERA5wt or rSERA5-A, produced elution profiles identical to that of the mock-digested substrate in the top panel (not shown). The intact substrate eluted as a weakly fluorescent peak at 11.2 min, whilst in the fully digested samples a major fluorescent peak appeared at 7.8 min. Identities of the indicated peaks, including the predicted and measured masses of the major digestion product as determined by electrospray mass spectrometry, are shown. The minor fluorescent digestion product eluting at 7.02 min could not be identified, but presumably is a result of less frequent cleavage at another position in the substrate peptide backbone.

B. Schematic structure of Abz-LAQAVRSSSR-Dpa (Jin *et al.*, 2002). In the intact peptide the fluorescence of the N-terminal donor Abz group is quenched by the accepting Dpa group. Cleavage at any point in the peptide backbone produces a highly fluorescent N-terminal product. Calculated molecular masses of the intact substrate and the Abz-LAQAVR major cleavage product obtained in this study are indicated.

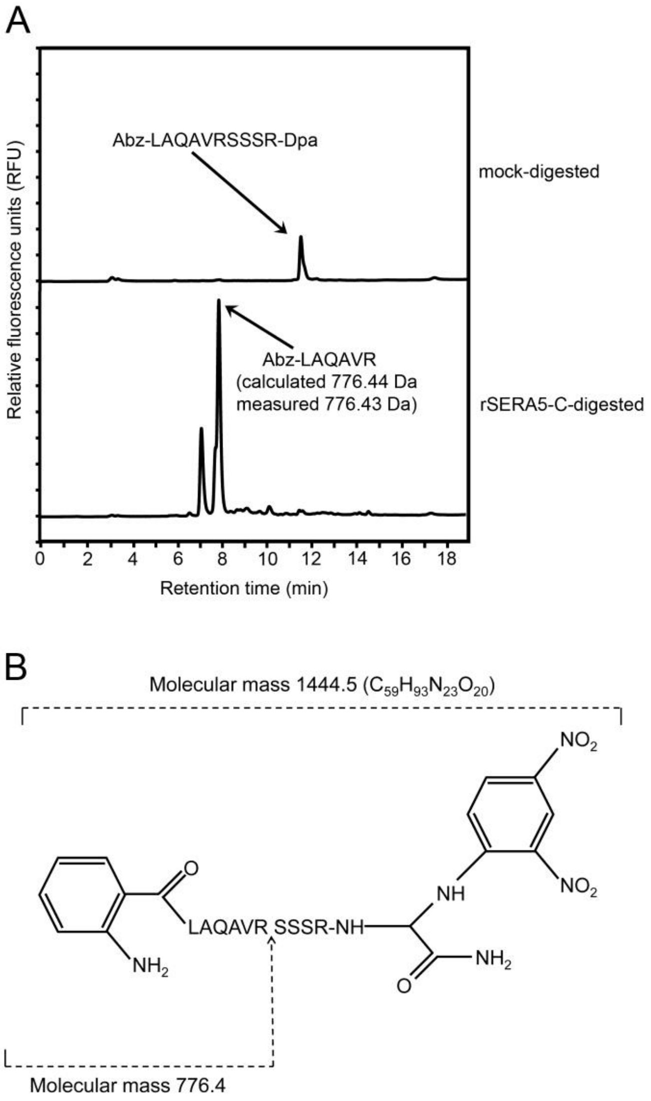
A
Abz-LAQAVRSSSR-Dpa
mock-digested
Abz-LAQAVR
(calculated 776.44 Da
measured 776.43 Da)
rSERA5-C-digested
Relative fluorescence units (RFU)
0 2 4 6 8 10 12 14 16 18
Retention time (min)
B
Molecular mass 1444.5 ($C_{59}H_{93}N_{23}O_{20}$)
NO_2
O
NH
NO_2
LAQAVR SSSR-NH
NH_2
NH_2
O
Molecular mass 776.4

Fig. S4. Cleavage of TACE substrate IV by rSERA5-C displays Michaelis-Menten kinetics.

A. Extended progress curve showing hydrolysis of Abz-LAQAVRSSSR-Dpa by rSERA5-C. Substrate (1.25 μM) in 100 μl 25 mM Bis-Tris buffer pH 6.5 containing 1 mM EDTA, 10 mM DTT and 0.05% (w/v) Tween 20, was supplemented with 1 μg purified rSERA5-C and incubated at 21°C. Increase in fluorescence was continuously monitored with time (at 5 min intervals) as described previously (Blackman *et al.*, 2002) using a Cary Eclipse fluorescence spectrophotometer (Varian) equipped with a 96-well microplate reader accessory (λ_{ex} 320 nm, λ_{em} 420 nm). The curve displayed represents individual reads from a single well of a kinetic assay; readings taken from triplicate wells in the same experiments showed no more than 4% variation from the plot shown at individual time-points.

B. Plot of initial hydrolysis rate (V) against substrate concentration. Initial rates were calculated under conditions in which <10% of substrate had been hydrolysed, based on progress curves such as that shown in (A).

C. Lineweaver-Burk transformation of the data shown in (B). The line of best fit was calculated by linear regression in Microsoft Excel. The intersect of the plot with the y-axis provides a measure of V_{max} (the hydrolysis rate under conditions of substrate saturation, calculated as 8.8 RFU min^{-1}), whilst the slope of the plot equates to K_m/V_{max}. The K_m value (the substrate concentration at which the hydrolysis rate is equal to half of V_{max}) is calculated in this experiment as 11.3 μM.

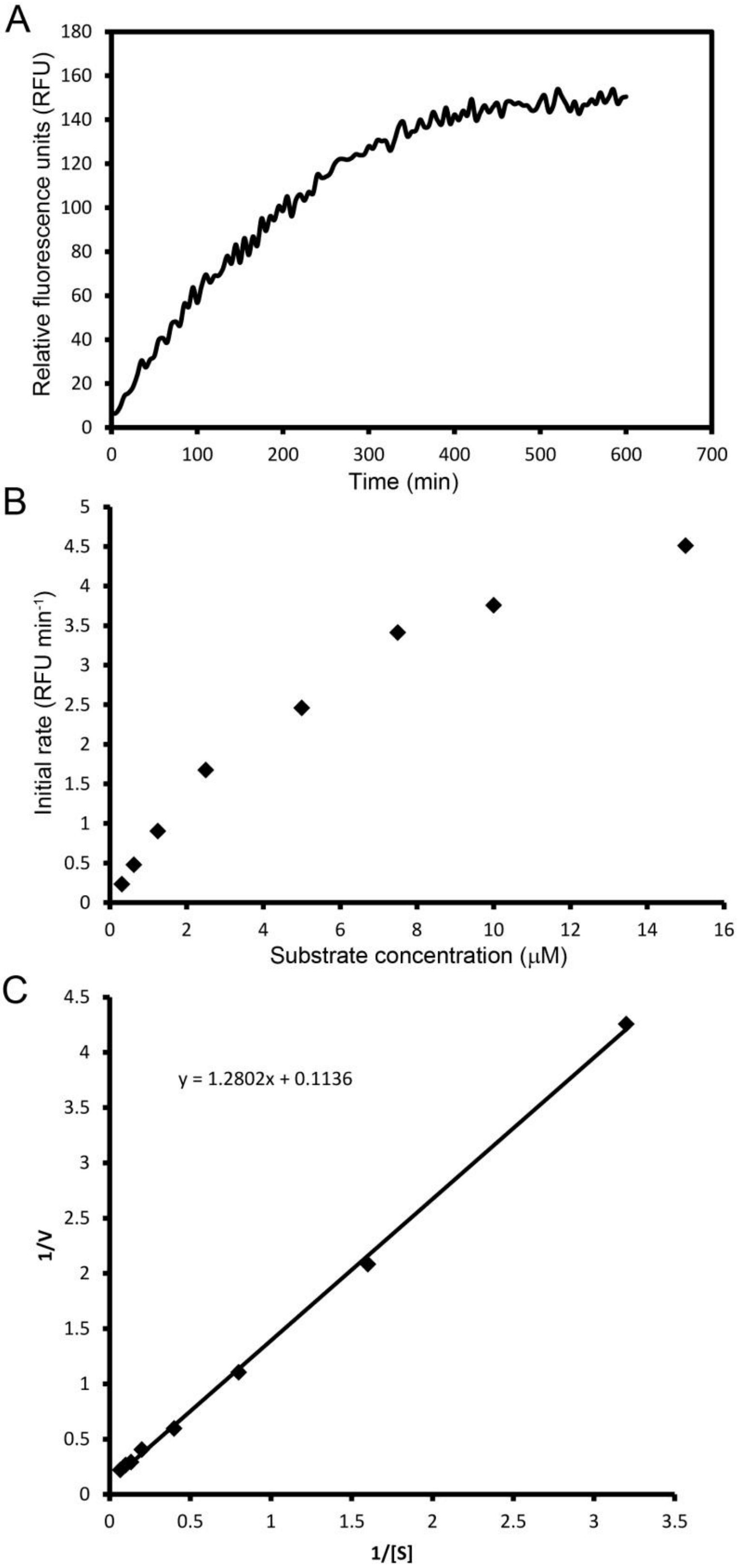
A
Relative fluorescence units (RFU)
Time (min)
0
20
40
60
80
100
120
140
160
180
0
100
200
300
400
500
600
700
B
Initial rate (RFU min^{-1})
Substrate concentration (μM)
0
0.5
1
1.5
2
2.5
3
3.5
4
4.5
5
0
2
4
6
8
10
12
14
16
C
y = 1.2802x + 0.1136
1/V
1/[S]
0
0.5
1
1.5
2
2.5
3
3.5
4
4.5
0
0.5
1
1.5
2
2.5
3
3.5

Fig. S5. Genomic integration by single-crossover homologous recombination of constructs targeting the *SERA5* locus.

A. Southern blot analysis of genomic DNA isolated from uncloned parasite cultures transfected with pHH1SERA5chimWT or pHH1SERA5chimS596A (3 independently-transfected cultures in each case, labelled 1-3) at drug-selection cycle 0 or cycle 4, compared with DNA from the parental 3D7 clone (control). By cycle 4, almost quantitative integration of the constructs was evident. Positions of migration of the 8.8 kb signal diagnostic of the endogenous *SERA5* locus, and the 4.4 and 5.7 kb signals characteristic of the integrant *SERA5* locus (see Fig. 3A, main manuscript) are indicated. The probe used for Southern analysis also detected an ~ 1 kb EcoRV fragment (indicated) derived from the input integration construct.

B. Southern blot analysis of chimS596A and chimWT clones obtained by limiting dilution, compared with the uncloned chimS596A transfection culture 2 (from drug cycle 0 and 4). All isolated clones except clone i show loss of the endogenous fragment signal at 8.8 kb and display the 4.4 and 5.7 kb bands characteristic of the integrant *SERA5* locus. In the examples shown, residual input integration construct was also detectable in DNA from the chimWT clones; this was an occasional feature of clones derived from all transfection experiments (not shown).

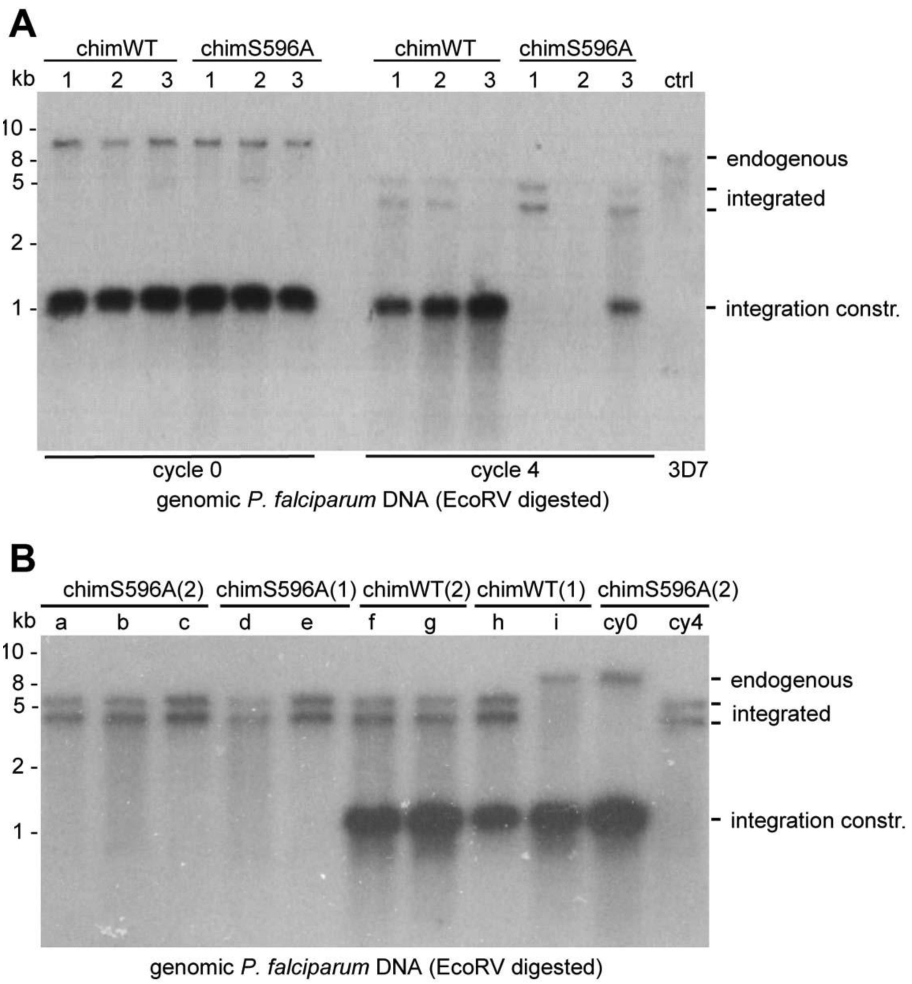
A
chimWT
chimS596A
chimWT
chimS596A
kb
1 2 3 1 2 3 1 2 3 1 2 3 ctrl
10 -
8 -
5 -
2 -
1 -
endogenous
integrated
integration constr.
cycle 0
cycle 4
3D7
genomic P. falciparum DNA (EcoRV digested)
B
chimS596A(2)
chimS596A(1)
chimWT(2)
chimWT(1)
chimS596A(2)
kb
a b c d e f g h i cy0 cy4
10 -
8 -
5 -
2 -
1 -
endogenous
integrated
integration constr.
genomic P. falciparum DNA (EcoRV digested)

Fig. S6. Reverse transcriptase-PCR analysis detects mRNA expression only from the chimeric *SERA5* ORF produced following genomic integration of targeting constructs.

A. RT-PCR was performed using total RNA purified from 3D7 parental *P. falciparum,* or various drug-resistant parasite clones obtained following transfection of the constructs described in Fig. 3A of the main manuscript. Primer pairs used (indicated) were designed to detect either mRNA transcribed from the chimeric *SERA5* locus resulting from the integration event depicted in Fig. 3A (primers +27 plus -11), or an mRNA transcript similar to that from the endogenous gene, which could potentially arise by extended splicing of run-through transcript of the modified locus (primers +27 plus -25). Parasite clones used as a source of RNA were: clone i (transfected with pHH1SERA5chimWT but displaying no integration of this construct; see Fig. S5B above); clone h (chimWT); and clone d (chimS596A). The results showed that primers +27 plus -25 produced an RT-PCR product only from parasites harbouring an unmodified *SERA5* locus, indicating that the unlikely possibility of run-through transcription and splicing to produce a wild-type mature *SERA5* mRNA transcript did not take place in parasites harbouring the chimeric *SERA5* locus. As expected, primers +27 plus -11 amplified a product only from the clones that had integrated the pHH1-based constructs. PCR reactions performed without reverse transcriptase (RT) usually gave no product. Where trace product is evident, this is likely due to the fact that the RNA used in these experiments was not DNase-treated before the RT reaction. However, because of the presence of intron sequence the products derived from these trace amounts of genomic DNA template migrate at a higher molecular weight than the mRNA-derived products. M, molecular mass marker DNA ladder (SmartLadder, Eurogentec). The primer pairs used for RT-PCR, and the predicted sizes of the resulting amplicons (which lack the introns present in the genomic sequence) are indicated.

B. RT-PCR products obtained from parasite clones h (chimWT) and d (chimS596A) were incubated in the absence or presence of the restriction enzyme BstUI. The restriction fragment pattern obtained is consistent with the presence of an additional BstUI site in the transcript arising from the chimS596A gene compared to that from the chimWT gene (see *Experimental Procedures*),

confirming preservation of the mutation at the mRNA level. M, 100 bp molecular mass marker DNA ladder.

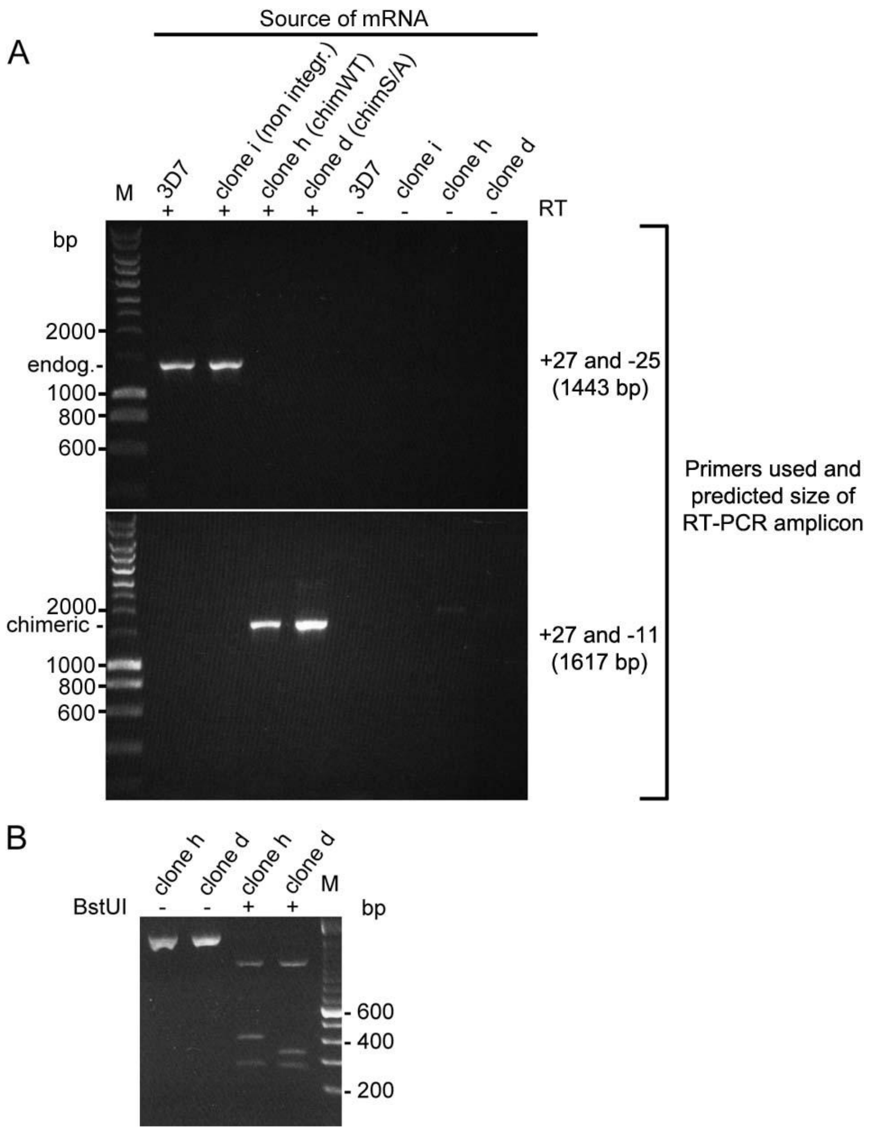

Fig. S7. Predicted structural consequences of substituting the SERA5 Ser596 with Arg.

Depicted are views facing the 'active site cleft' of the *P. falciparum* SERA5 papain-like domain (PDB ID: 2WBF) represented as a green molecular surface, with Ser596 shown in red. The wild-type protein is depicted on the left-hand side, whilst the predicted structure resulting from substitution of Ser596 with Arg is shown on the right. The much larger Arg side chain (shown as blue sticks with its surface as a blue mesh) is predicted to extend into the cleft and interfere with binding of putative client molecules. The figures were prepared with PyMol (http://pymol.org/).

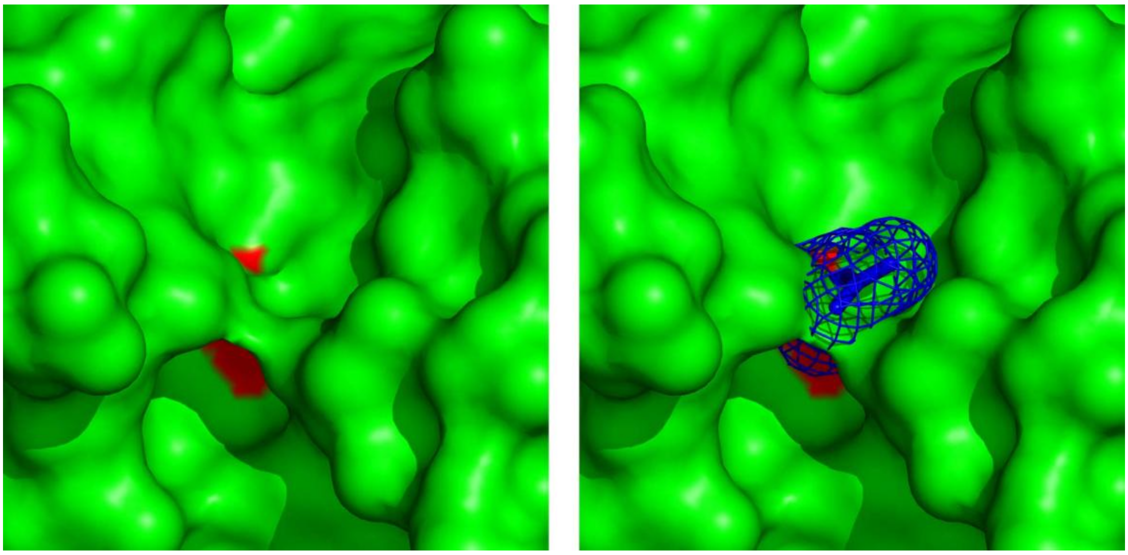

Fig. S8. Introduction of a mini TAP tag into the endogenous *P. falciparum SERA5* gene.

Schematic depiction of the primary sequence modifications made in introducing the mini TAP tag into the *SERA5* gene using transfection construct pHH1SERA5chimΔP6mTAP. The modification replaces the bulk of the P6 encoding region (see Fig. 1B of the main manuscript) with the mini TAP tag sequence. The wild type 3D7 SERA5 sequence (Lys836-Thr900) is shown aligned with the sequence introduced by integration of pHH1SERA5chimΔP6mTAP. Non-wild type residues introduced are shown in red, with the major elements of this underlined and indicated. Note that introduction of the mini TAP tag sequence modifies the PfSUB1 site 2 cleavage site. Whether this affects PfSUB1-mediated cleavage at his position was not examined in this study.

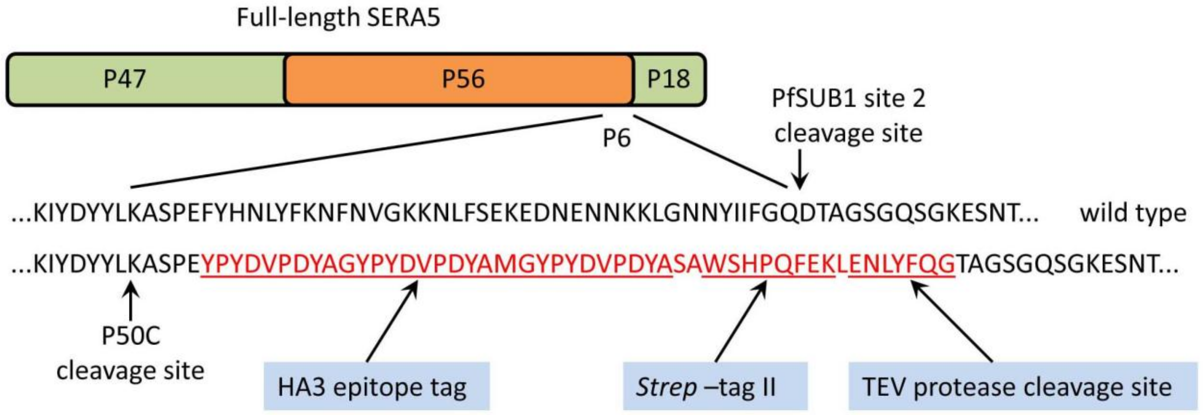

Table S1. Peptide substrates used to examine the protease activity of purified native *P. falciparum* P50 and recombinant SERA5 (rSERAwt, rSERA5-A and rSERA5-C).

Substrate name	Substrate structure	Cleavage determination[j,k]
PfSUB2 PD	NH_2-HEENNKHKLNKKNSSNYKNDKSLDEC-OH[a]	RP-HPLC
PfSUB3	NH_2-RNKLYLKKKKECSNYNTSNDGHGHC-OH[a]	RP-HPLC
ST-24	NH_2-IKSKKTYINSNISNKWKKKSRYLHC-OH[a]	RP-HPLC
PfAMA1stub	NH_2-SNNEVVVKEEYKDEYADIPEHKPTC-OH[a]	RP-HPLC
Chymotrypsin substrate	Suc-AAPF-AMC[b,c,d,e,f,g,h]	Fluorometric (Ex 360 nm, Em 460 nm)
Chymotrypsin substrate	Suc-LLVY-AMC[b,c,d,e,f,g,h]	Fluorometric (Ex 360 nm, Em 460 nm)
pepF1-6R	Ac-C(Rho)LVSADNIDIC(Rho)-OH[d,e,i]	Fluorometric (Ex 552, Em 580 nm)
SERA5st1F-6R	Ac-C(Rho)IKAETEDDC(Rho)-OH[d,i]	Fluorometric (Ex 552, Em 580 nm)
SERA5st2F-6R	Ac-C(Rho)IFGQDTAGC(Rho)-OH[d,i]	Fluorometric (Ex 552, Em 580 nm)
SERA4st1F-6R	Ac-C(Rho)ITAQDDEEC(Rho)-OH[e,i]	Fluorometric (Ex 552, Em 580 nm)
SERA4st1F-6R12	Ac-C(Rho)KITAQDDEESC(Rho)-OH[e,i]	Fluorometric (Ex 552, Em 580 nm)
SERA4st1F-6RprimeS	Ac-C(Rho)ITAQSDEEC(Rho)-OH[e,i]	Fluorometric (Ex 552, Em 580 nm)
Papain substrate	Nα-Benzoyl-L-Arg-AMC[d]	Fluorometric (Ex 360 nm, Em 460 nm)
Caspase-3 substrate	Ac-DEVD-AMC[d]	Fluorometric (Ex 360 nm, Em 460 nm)
Caspase-3 substrate	Ac-DMQD-AMC[f,g,h]	Fluorometric (Ex 360 nm, Em 460 nm)
Caspase-6 substrate	Ac-VEID-AMC[f,g,h]	Fluorometric (Ex 360 nm, Em 460 nm)
Caspase-1 substrate	Ac-WEHD-AMC[f,g,h]	Fluorometric (Ex 360 nm, Em 460 nm)
Proteasome substrate	Z-GGL-AMC[f,g,h]	Fluorometric (Ex 360 nm, Em 460 nm)
Factor IX/Xa substrate	Boc-IEGR-AMC[f,g,h]	Fluorometric (Ex 360 nm, Em 460 nm)
SUB1trunc substrate	Ac-KITAQ-AMC[f,g,h]	Fluorometric (Ex 360 nm, Em 460 nm)
Furin substrate	Boc-RVRR-AMC[d,f,g,h]	Fluorometric (Ex 360 nm, Em 460 nm)
SUB2trunc substrate	Z-GML-AMC[f,g,h]	Fluorometric (Ex 360 nm, Em 460 nm)
GR-AMC	NH_2-GR-AMC[f,g,h]	Fluorometric (Ex 360 nm, Em 460 nm)
Cathepsin K substrate	Z-LR-AMC[f,g,h]	Fluorometric (Ex 360 nm, Em 460 nm)
Cathepsin B substrate V	Abz-GIVRAK(Dnp)-OH[f,g,h]	Fluorometric (Ex 320 nm, Em 420 nm)
β-secretase substrate VII	Abz-VKMDAE-EDDnp[f,g,h]	Fluorometric (Ex 320 nm, Em 420 nm)
TACE substrate IV	Abz-LAQAVRSSSR-Dpa[f,g,h]	Fluorometric (Ex 320 nm, Em 420 nm)

[a-h]Assay buffer conditions: [a]25 mM HEPES pH 6.5, 12.5 mM CHAPS, 10 mM $CaCl_2$; [b]25 mM HEPES pH 6.5, 1 mM DTT; [c]25 mM HEPES pH 6.5, 1 mM DTT, 20 mM CHAPS, 1.5 mM $CaCl_2$; [d]50 mM HEPES pH 6.5, 25 mM CHAPS, 10 mM $CaCl_2$, 1 mM DTT; [e]25 mM HEPES pH 7.4, 25 mM CHAPS, 12 mM $CaCl_2$; [f]25 mM HEPES pH 7.4, 15 mM $CaCl_2$, 2 mM DTT; [g]25 mM Bis-Tris pH 6.5, 1 mM EDTA, 5 mM DTT; [h] 50 mM sodium acetate pH 5.5, 5 mM $MgCl_2$, 5 mM DTT.

[i] C(Rho), terminal Cys residues labelled with 6-iodoacetamido tetramethylrhodamine, produced as described previously (Blackman *et al.*, 2002, Koussis *et al.*, 2009, Yeoh *et al.*, 2007, Withers-Martinez *et al.*, 2012).

[j] Peptide hydrolysis was assessed by fractionation of digestion reactions on a Vydac 4.6 mm × 25 cm C_{18} reversed-phase column, eluted at 1 ml min^{-1} with a 0-55% (v/v) gradient of acetonitrile in 0.1% (v/v) TFA over 60 min.

[k] Fluorogenic peptide hydrolysis was monitored continuously at room temperature as previously described (Blackman *et al.*, 2002) using a Varian Cary Eclipse fluorescence spectrophotometer by measuring the increase in fluorescence of AMC or Abz using the indicated excitation and emission wavelengths.

Table S2. Oligonucleotide primers used in this study.

**Primer name**	**Sequence (5'-3')**
+S5endogHpaI	GGGGTTAACGTACGGGAACAGTTAGAGGAG
-S5endogClaI	TATTATCGATAGATTCTGTTAATTTATATTCAG
+S5Seq1021	GCGATATTCCGGAAAAATGCG
-S5stopXhoI	ACTCGAGCTACACGTAGCAGAAATAGCATTC
-S5_StoA_BstUI	CGCAAAAATCCACGCGGTATCGCAGTT
+S5_StoA_BstUI	AACTGCGATACCGCGTGGATTTTTGCG
-S5XbaIKO	TAAGAAACCATAATTCTAGATAATCTGCAGAA
+S5XbaIKO	TTCTGCAGATTATCTAGAATTATGGTTTCTTA
-S5StoR_MluI	CTCGCAAAAATCCAACGCGTATCGCAGTTG
+S5StoR_MluI	CAACTGCGATACGCGTTGGATTTTTGCGAG
+S5Seq2021	GAAAGTGGGCGAACAGTG
-1longHS	GAACGTCGTAAGGGTAGCCCATGGCATAGTCCGGGACGTCATAGGGATAGCCCGCATAGTCAGGA ACATCGTAAGGGTACTCCGGGCTCGCCTTCAGATAATAATC
+2longHS	TGGGCTACCCTTACGACGTTCCAGATTACGCTAGCGCTTGGAGCCACCCGCAGTTCGAGAAACTTGA GAATTTGTATTTTCAGGGTACAGCGGGTAGCGGCCAGA
-29_S5stopXhoI	GGGTACTCGAGCTACACGTAGCA
+BamHImelit	CCCGGATCCATGAAATTCTTAGTCAACGTTGCC
-melitSERA5	GCCGGTCTGGCTTTCGCCGGTGGCATAGATGTAAGAAAT
-S5ENDHindIII	CCCAAGCTTTCACACGTAGCAGAAATAGCATTC
-S5_StoC	CGCAAAAATCCAGCAGGTATCGCAGTT
+ S5_StoC	AACTGCGATACCTGCTGGATTTTTGCG
+27	CAATATCATTTGAATCAAACAGTGGT
-11	CTTTGCCATCCAGGCTGTTC
-25	CCATTGGACTAGAACCTTCAT
b	ACTACGCAATAGGATCAGAC
Q+SERA1[a]	GCGTATCAAGAGATACTCTAGATAAGGATAG
Q-SERA1[a]	CTGATATCTACTTTGTTATTTGTAACTCCTG
Q+SERA2[a]	TTTATATTGTATCTTCAACATGATTTGATAAC
Q-SERA2[a]	TAAATCTACTTGACCTGGAGCTATCC
Q+SERA3[a]	AAGTATGTTAAAGAATAAATGTGAATCTGG
Q-SERA3[a]	TCTTCAGATTTTTGTGGAGTAGCAG
Q+SERA4[a]	TGTGAAAGATGACACAAAAAAATTTG
Q-SERA4[a]	ATTTCATCCACATCTTGTTCCTTG
Q+SERA5[a]	TATATGTGAAACTGGAAAAAACTTCAAG
Q-SERA5[a]	ACTTTCTTACATCAACTTTGTTATTTTCAG
Q+SERA6[a]	GTTGAGCACAAGGGGAACTTC
Q-SERA6[a]	CTTACATCCGAATCTTCACTCTTTG
Q+SERA7[a]	GGTCCATCCGATTTAATAGATATAAATG
Q-SERA7[a]	TCGATGTTTGTTGGTTTAGAATCC
Q+SERA8[a]	CCTTGTACACAAAACCATGAAAGC
Q-SERA8[a]	CGTGTAAACCCAACGTATGCC
Q+SERA9[a]	AAGGATCAACTGAATATGAAGAAAGTG
Q-SERA9[a]	TCTAATGTACTATTTAAGTCTTCAAAACTTATC
Q+tubulin[a]	ATGAGCATGGAATAGATCCAAGTG
Q-tubulin[a]	CCAGGTTCCAAGTCCATCAATATAG
Q+AMA1[a]	CCTCCAACAGAACCTCTTATGTCAC
Q-AMA1[a]	TCCTGCATGTCTTGAACATAAAGTC
Q+MSP1[a]	CTACGAAATCTGTTCAAATACCAAAAG
Q-MSP1[a]	TTCTTTAGTATGAGGATTCATTAAATCAC

[a] All primers with names beginning with 'Q' were used for the RTqPCR analyses.

References

Blackman, M.J., J.E. Corrie, J.C. Croney, G. Kelly, J.F. Eccleston & D.M. Jameson, (2002) Structural and biochemical characterization of a fluorogenic rhodamine-labeled malarial protease substrate. *Biochemistry* **41**: 12244-12252.

Debrabant, A., P. Maes, P. Delplace, J.F. Dubremetz, A. Tartar & D. Camus, (1992) Intramolecular mapping of Plasmodium falciparum P126 proteolytic fragments by N-terminal amino acid sequencing. *Molecular and biochemical parasitology* **53**: 89-95.

Jin, G., X. Huang, R. Black, M. Wolfson, C. Rauch, H. McGregor, G. Ellestad & R. Cowling, (2002) A continuous fluorimetric assay for tumor necrosis factor-alpha converting enzyme. *Anal Biochem* **302**: 269-275.

Koussis, K., C. Withers-Martinez, S. Yeoh, M. Child, F. Hackett, E. Knuepfer, L. Juliano, U. Woehlbier, H. Bujard & M.J. Blackman, (2009) A multifunctional serine protease primes the malaria parasite for red blood cell invasion. *EMBO J* **28**: 725-735.

Li, J., T. Mitamura, B.A. Fox, D.J. Bzik & T. Horii, (2002) Differential localization of processed fragments of Plasmodium falciparum serine repeat antigen and further processing of its N-terminal 47 kDa fragment. *Parasitol Int* **51**: 343-352.

Withers-Martinez, C., C. Suarez, S. Fulle, S. Kher, M. Penzo, J.P. Ebejer, K. Koussis, F. Hackett, A. Jirgensons, P. Finn & M.J. Blackman, (2012) Plasmodium subtilisin-like protease 1 (SUB1): insights into the active-site structure, specificity and function of a pan-malaria drug target. *International journal for parasitology* **42**: 597-612.

Yeoh, S., R.A. O'Donnell, K. Koussis, A.R. Dluzewski, K.H. Ansell, S.A. Osborne, F. Hackett, C. Withers-Martinez, G.H. Mitchell, L.H. Bannister, J.S. Bryans, C.A. Kettleborough & M.J. Blackman, (2007) Subcellular discharge of a serine protease mediates release of invasive malaria parasites from host erythrocytes. *Cell* **131**: 1072-1083.
